# Activation of STAT3 through combined SRC and EGFR signaling drives resistance to a mitotic kinesin inhibitor in glioblastoma

**DOI:** 10.1016/j.celrep.2022.110991

**Published:** 2022-06-21

**Authors:** Rajappa S. Kenchappa, Athanassios Dovas, Michael G. Argenziano, Christian T. Meyer, Lauren E. Stopfer, Matei A. Banu, Brianna Pereira, Jessica Griffith, Afroz Mohammad, Surabhi Talele, Ashley Haddock, Natanael Zarco, William Elmquist, Forest White, Vito Quaranta, Peter Sims, Peter Canoll, Steven S. Rosenfeld

**Affiliations:** 1 Department of Cancer Biology, Mayo Clinic, Jacksonville, FL 32224, USA; 2 Department of Pathology and Cell Biology, Columbia University Irving Medical Center, New York, NY 10032, USA; 3 Department of Biochemistry, Vanderbilt University, Nashville, TN 37232, USA; 4 Department of Biological Engineering, Massachusetts Institute of Technology, Cambridge, MA 02139, USA; 5 Department of Neurosurgery, Mayo Clinic, Jacksonville, FL 32224, USA; 6 Department of Pharmaceutics, University of Minnesota, Minneapolis, MN 55455, USA; 7 Department of Systems Biology, Columbia University Irving Medical Center, New York, NY 10032, USA; 8 Lead contact

## Abstract

Inhibitors of the mitotic kinesin Kif11 are anti-mitotics that, unlike vinca alkaloids or taxanes, do not disrupt microtubules and are not neurotoxic. However, development of resistance has limited their clinical utility. While resistance to Kif11 inhibitors in other cell types is due to mechanisms that prevent these drugs from disrupting mitosis, we find that in glioblastoma (GBM), resistance to the Kif11 inhibitor ispinesib works instead through suppression of apoptosis driven by activation of STAT3. This form of resistance requires dual phosphorylation of STAT3 residues Y705 and S727, mediated by SRC and epidermal growth factor receptor (EGFR), respectively. Simultaneously inhibiting SRC and EGFR reverses this resistance, and combined targeting of these two kinases *in vivo* with clinically available inhibitors is synergistic and significantly prolongs survival in ispinesib-treated GBM-bearing mice. We thus identify a translationally actionable approach to overcoming Kif11 inhibitor resistance that may work to block STAT3-driven resistance against other anti-cancer therapies as well.

## In brief

Inhibitors of the mitotic kinesin Kif11, such as ispinesib, have been ineffective anti-cancer therapies because of the development of resistance. Kenchappa et al. find that ispinesib resistance in glioblastoma is due to phosphorylation-mediated activation of STAT3 and that blocking phosphorylation with FDA-approved drugs reverses this resistance and renders ispinesib effective.

## Graphical Abstract

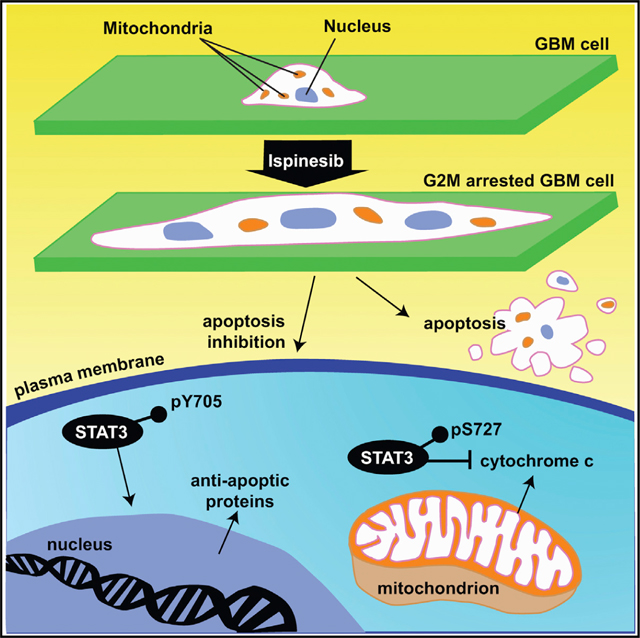

## INTRODUCTION

The outcome for patients afflicted with glioblastoma (GBM), the most common and malignant of primary brain tumors in adults, has changed little despite decades of research (Puduvalli and Hoang, 2018). While a number of signaling pathways have been shown to drive GBM tumorigenesis, and while CNS permeant inhibitors of several of these pathways have been identified, the efficacy of these drugs has been disappointing (De Witt Hamer, 2010; Reardon et al., 2015; Szerlip et al., 2012). This failure likely reflects redundancy in oncogenic signaling pathways (Dong et al., 2020; Olmez et al., 2018), allowing tumor cells to compensate for loss of one pathway by activating another. Targeting the cellular components where these pathways converge might avoid this problem. One of these points of convergence is the microtubule-based cytoskeleton, which drives tumor cell invasion and proliferation (Wood et al., 2001; Canta et al., 2009). Although drugs disrupting microtubule dynamics, including vinca alkaloids and taxanes, have been used successfully against a variety of malignancies (Wood et al., 2001), their dose-limiting neurotoxicities and poor CNS penetration limit their efficacy in GBM. An alternative approach would be to target mitotic kinesins, molecular motor proteins that use microtubules to organize the mitotic spindle. An extensive array of inhibitors against one of these, the mitotic kinesin Kif11, has been developed, and as expected, they are not neurotoxic (Rath and Kozielski, 2012; Rosenfeld, 2015). Furthermore, we have shown that one of them, ispinesib, is highly CNS penetrant and prolongs survival in murine GBM models (Gampa et al., 2020; Venere et al., 2015).

Unfortunately, clinical trials of Kif11 inhibitors in hematologic and solid malignancies have been disappointing (Rath and Kozielski, 2012; Rosenfeld, 2015). These drugs are only effective against cells in the G_2_M phase of the cell cycle, and since most of them have a short half-life, the probability of a drug affecting a tumor cell when it is vulnerable is low (Komlodi-Pasztor et al., 2012). While the Kif11 inhibitor filanesib has a long half-life and is effective against recurrent multiple myeloma, relapse with this drug still occurs, implying that even with more favorable pharmacokinetics, treatment failure ultimately reflects the development of resistance. Prior studies have identified four resistance mechanisms: (1) point mutations in Kif11 that block drug binding (Kasap et al., 2014); (2) upregulation of Kif15, which can compensate for loss of Kif11 (Sturgill and Ohi, 2013); (3) upregulation of the epidermal growth factor receptor (EGFR), which enables normal levels of Kif15 to drive mitotic progression (Mardin et al., 2013); and (4) upregulation of drug efflux transporters (Gampa et al., 2020). What is not known, however, is if there are other mechanisms of resistance, in what cell types these occur, and if resistance can be reversed with clinically available drugs. In this study, we find that development of ispinesib resistance in GBM occurs by a mechanism not previously described for Kif11 inhibitors, and we show that resistance can be reversed with clinically available CNS permeant drugs.

## RESULTS

### Once established, ispinesib resistance in GBM is long lasting and associated with mitotic defects

**Figure 1.**
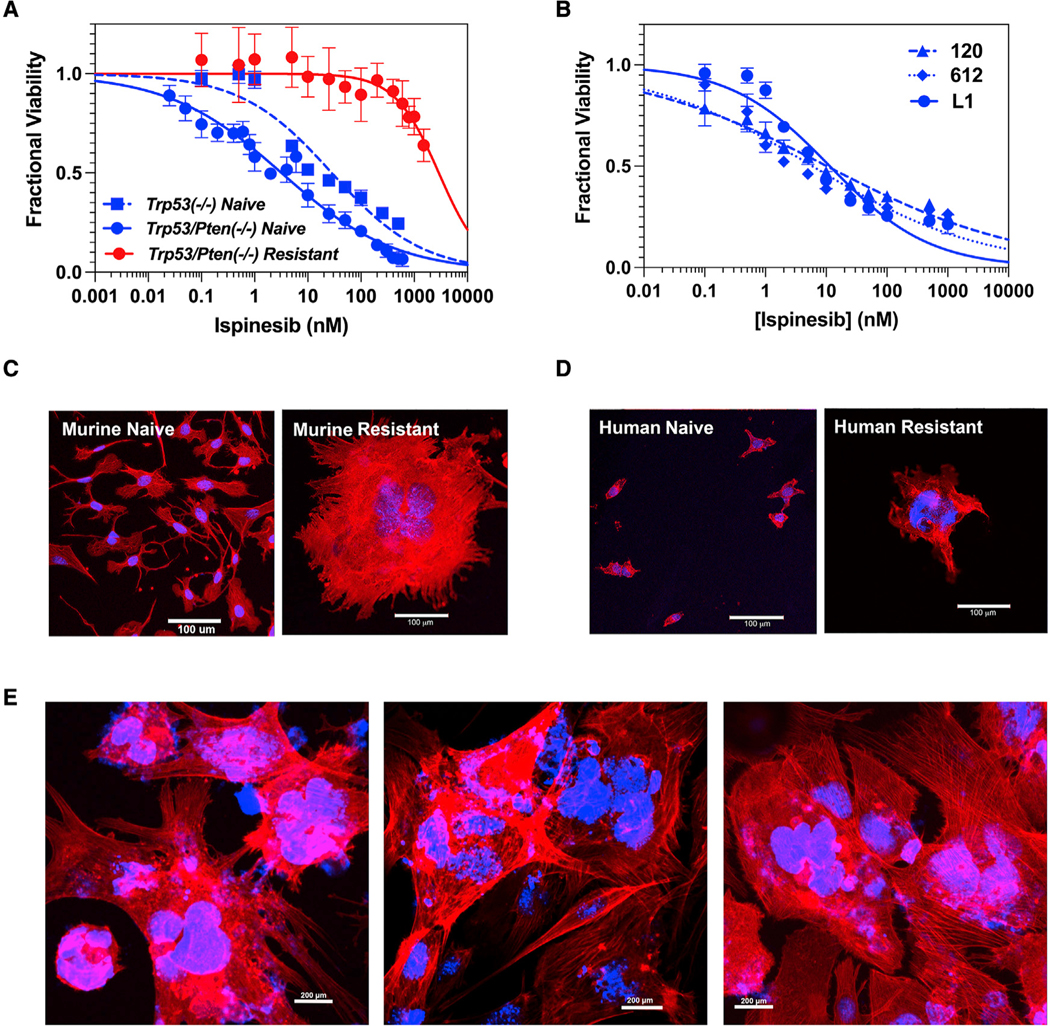
Effects of ispinesib on viability and morphology of murine and human GBM cells (A) Ispinesib dose-response curves for ispinesib-naive murine *Trp53*^−/−^ (dashed blue) and *Trp53/Pten*^−/−^ (solid blue) GBM cells and ispinesib-resistant *Trp53/Pten*^−/−^ GBM cells (red; see also Table S1). (B) Ispinesib dose-response curves for three human GBM cell lines (see also Table S1). For all dose-response curves, each point represents the mean ± 1 SD of 8 replicates. (C and D) Ispinesib-naive and -resistant murine *Trp53/Pten*^−/−^ (C) and human L1 (D) GBM cells stained with rhodamine phalloidin for F actin (red) and DAPI for nuclei (blue). Scale bar: 100 μm. (E) Low power images of three randomly selected fields of the murine resistant cells from (C) demonstrate that nearly all cells assume an enlarged, multinucleated morphology. Scale bar: 200 μm.

We examined the ispinesib sensitivity of two proneural murine GBM lines derived from genetically engineered mouse models (GEMMs)—one deleted for *Trp53*, and the other co-deleted for *Trp53* and *Pten*, referred to as *Trp53*^−/−^ and *Trp53/Pten*^−/−^, respectively (Lei et al., 2011). Lesions in these two tumor suppressors are among the most common mutations in human GBM. Dose-response curves for ispinesib sensitivity for these two cell lines (Figure 1A, solid and dashed blue) could be fit to a Hill equation, revealing low nanomolar values of half maximal effective concentration (EC_50_) (Table S1). We made *Trp53/Pten*^−/−^ cells resistant to ispinesib by culturing them in the presence of 75 nM of the drug for 3 weeks. These cells retain their ispinesib resistance, with a >1,000-fold increase in EC_50_ (Figure 1A, red). Three primary human GBM cell lines (L1, 612, and 120) are likewise highly sensitive to this drug (Figure 1B; Table S1). We generated an ispinesib-resistant human L1 GBM derivative line by culturing in ispinesib as above. In both murine and human GBM, resistance is associated with formation of giant, polyploid cells (Figures 1C–1E). These features can be seen in cells that have undergone a G_2_M block, and consistent with this, we find that resistant cells upregulate expression of phospho-histone H3 (Figure S1). While this block is typically followed by apoptotic cell death (Vakifahmetoglu et al., 2008), this clearly is not occurring. The four previously described ispinesib resistance mechanisms each work by preventing the drug from disrupting spindle function and inducing a subsequent G_2_M arrest, and these resistance mechanisms therefore cannot explain how resistance develops in murine or human GBM cells.

### In *Trp53/Pten*^−/−^ GBM cells, ispinesib resistance cannot be explained by any of the previously described mechanisms

**Figure 2.**
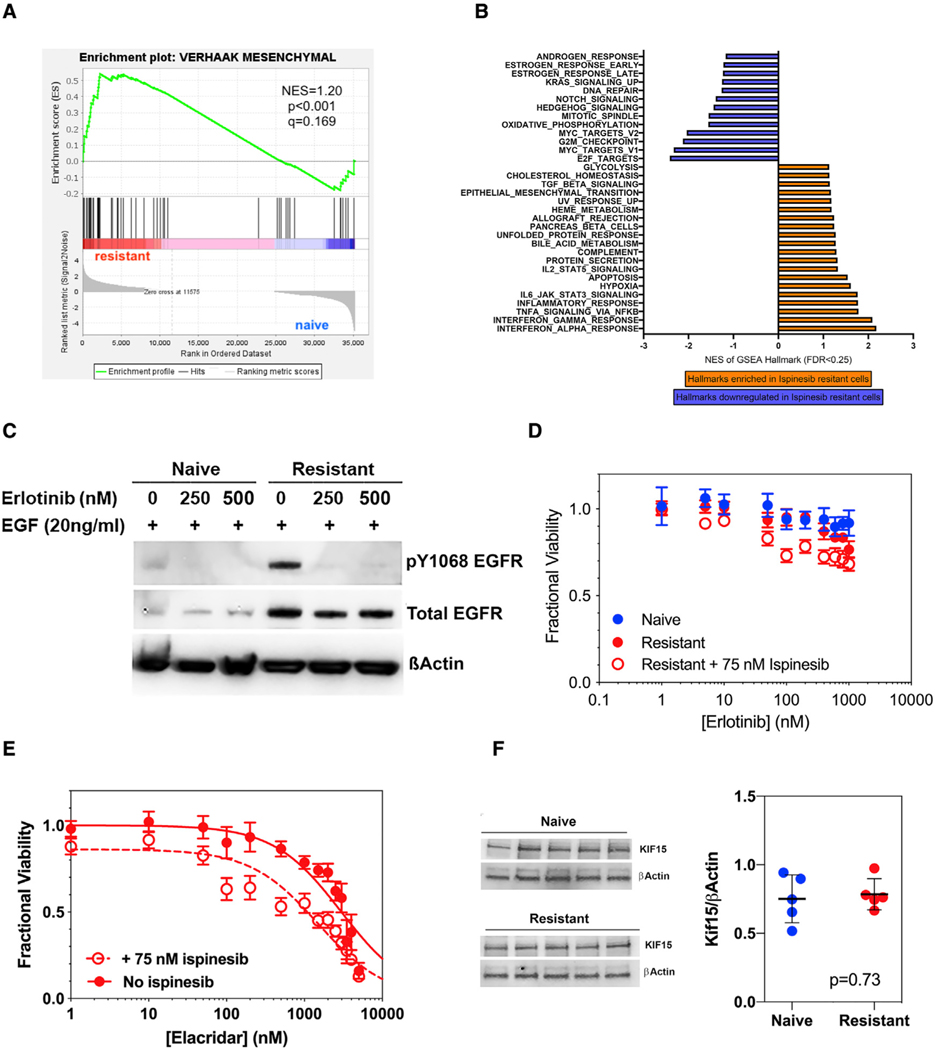
Transcriptional consequences of ispinesib resistance (A) Gene set enrichment analysis (GSEA) was performed on three biological replicates of ispinesib-naïve and -resistant *Trp53/Pten*^−/−^ cells and demonstrates that resistance is associated with a mesenchymal phenotype. (B) Gene Ontology analysis shows that resistance leads to downregulation in cell-cycle regulatory and mitotic gene signatures and upregulation in epithelial-mesenchymal transition, inflammatory, and apoptosis-related gene signatures. (C) Development of resistance is associated with significant upregulation of both total EGFR and active pY1068 EGFR. (D) Dose response of erlotinib treatment of *Trp53/Pten*^−/−^ cells that are drug naive (blue), drug resistant in the absence of ispinesib (solid red), or drug resistant in the presence of 75 nM ispinesib (open red). (E) Dose-response curves for treatment of ispinesib-resistant *Trp53/Pten*^−/−^ cells with the ABCB1/ABCG2 inhibitor elacridar (see also Table S1). For all dose-response curves, each point represents the mean ± 1 SD of 8 replicates. (F) (Left) Western blot of Kif15 in ispinesib-naive and -resistant *Trp53/Pten*^−/−^ cells. (Right) Normalized plot of Kif15 in naive (blue) and resistant (red) *Trp53/Pten*^−/−^ cells. p value calculated using a two tailed t test.

Resistance to ispinesib can result from point mutations that block drug binding, including S120, D130, and A133 in the human Kif11 sequence (Kasap et al., 2014). An additional mutation at G268 enables Kif11 to bundle microtubules, allowing even physiologically normal levels of Kif15 to drive spindle assembly (Sturgill et al., 2016). However, Sanger sequencing of the Kif11 gene from ispinesib-resistant murine *Trp53/Pten*^−/−^ cells shows no evidence of mutations in the corresponding residues in mouse sequence (S119, D129, A132, and G267). To examine more globally for mechanisms driving ispinesib resistance, we performed bulk RNA sequencing (RNA-seq) from three biological replicates of ispinesib-naive and -resistant *Trp53/Pten*^−/−^ cells, and this reveals >6,200 genes that are differentially expressed to a significant degree (adjusted p value [p_adj_] < 0.05), with 3,182 genes upregulated (Table S2, Up) and 3,038 genes downregulated (Table S2, Down). Ispinesib resistance is associated with acquisition of a mesenchymal phenotype (Figure 2A), and resistance leads to downregulation of cell-cycle regulatory and mitotic gene signatures and upregulation of epithelial-mesenchymal transition, inflammatory, and apoptosis-related gene signatures (Figure 2B). Analysis of the leading-edge genes for the apoptosis and interleukin-6 (IL-6)-JAK-STAT3 signaling ontologies reveals specific genes contributing to the enrichment of each ontology (Table S3).

Ispinesib resistance is associated with an ~3-fold upregulation of mRNA for EGFR (p_adj_ = 0.0012), along with upregulation of total EGFR protein and phosphorylated EGFR (Figure 2C). Several other receptor tyrosine kinases (RTKs) are also transcriptionally upregulated (summarized in Table S4), including PDGFRβ (2.7-fold). Erlotinib, a highly specific EGFR tyrosine kinase inhibitor, has no effect on ispinesib resistance (Figure 2D), despite reducing phosphorylated EGFR (Figure 2C). Another potential mediator of ispinesib resistance, the drug efflux transporter ABCB1, is upregulated 2.8-fold in resistant cells (p_adj_ =6 × 10^−7^). We treated ispinesib-naive and -resistant cell lines with elacridar, an inhibitor of the ABCB1 and ABCG2 efflux transporters. While elacridar by itself is toxic to ispinesib-resistant cells at high concentrations, its EC_50_ is reduced only ~2-fold in the presence of 75 nM ispinesib (Figure 2E; Table S1). Finally, our RNA-seq database also revealed that Kif15 is not significantly upregulated transcriptionally in resistant cells (p_adj_ = 0.25), and we confirmed this at the protein level as well (Figure 2F).

### Phosphoproteomics implicate SRC family kinases in the development of ispinesib resistance

**Figure 3.**
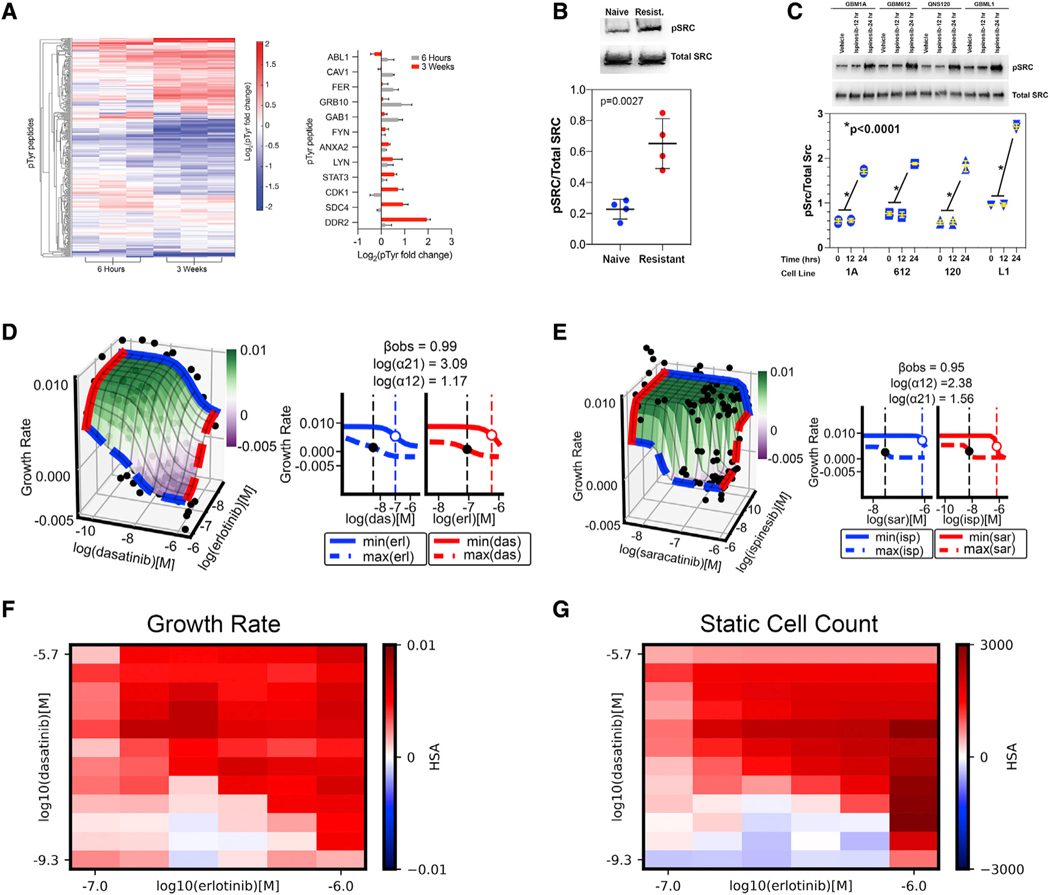
Ispinesib resistance depends upon the activities of SFKs and EGFR (A) (Left) Hierarchical clustering of unique tyrosine phosphorylated peptides (distance metric = Euclidean) of *Trp53/Pten*^−/−^ cells treated with 200 nM ispinesib for 6 h (left) or 75 nM ispinesib for 3 weeks (right). Values represent log_2_-fold change in tyrosine phosphorylation (pTyr) of each sample over the average signal of DMSO-treated control cells. (Right) Log_2_-fold change in pTyr of SRC family kinase and SFK substrates. Error bars represent SD for three biological replicates. (B) (Top) Immunoblot for total and phosphorylated SRC (pSRC) in *Trp53/Pten*^−/−^ naive and resistant cells. (Bottom) Plot of normalized pSRC in ispinesib-naive (blue) and -resistant (red) *Trp53/Pten*^−/−^ cells. Differences are statistically significant by two-tailed t test. (C) Immunoblots of total SRC and pSRC (top) and plot of normalized pSRC (bottom) in four human GBM cell lines (1A, 612, 120, and L1) treated with vehicle (t= 0) or with 50 nM ispinesib for 12 and 24 h. For each cell line, normalized pSRC significantly increases after 24 h of ispinesib exposure (p < 0.0001, pairwise two-tailed t test). (D) (Left) Dose-response surface for the combination of dasatinib and erlotinib for ispinesib-resistant *Trp53/Pten*^−/−^ cells. The growth rate for each pair of tested concentrations (black dots) was fit to the MuSyC equation (surface plot). The dose-response surface describes the relationship between the drug concentrations (x,y axis) and the growth rate constant (z axis, colored bar, expressed as h^−1^). (Right) Projected edges of the surface for the maximum and minimum tested in the combination. Vertical dotted lines mark the EC_50_ for each curve. Synergistic efficacy (β_obs_) is defined as the percentage increase in effect at the maximum tested doses of the combination over either drug alone. Log(α12) is the log-fold increase in potency of drug 1 (dasatinib) given the presence of drug 2 (erlotinib). Log(α21) is the log-fold increase of erlotinib’s potency due to dasatinib. (E) Analogous dose-response surface (left) and projected surface edges (right) for the combination of ispinesib and saracatinib. (F and G) Highest single agent (HSA) quantification of drug-drug interactions. HSA is equal to the most efficacious single agent minus the effect of combinations for each dose pair. HSA > 0 (red) means the growth rate is lower in the combination than either single dose (e.g., synergistic). The heatmap depicts the mean HSA values for each dose pair of the erlotinib/saracatinib drug combination against ispinesib-resistant *Trp53/Pten*^−/−^ cells. Drug effect was measured using cell growth rate (F) and static cell counts (G) after 96 h of treatment.

Since responses to therapy can also be modulated by post-translational modifications, we treated ispinesib-naive *Trp53/Pten*^−/−^ cells with vehicle or with ispinesib for 6 h or for 3 weeks and performed phosphotyrosine proteomics. We found increased tyrosine phosphorylation in multiple peptides as early as 6 h following treatment, which becomes significantly more pronounced after 3 weeks (Figure 3A, left). Many of these peptides are associated with Src family kinase (SFK) activity (Figure 3A, right), including phosphorylation of Fyn and Lyn kinases and multiple SFK substrates, including the transcription factor STAT3 (Aznar et al., 2001; Yu et al., 2014; Wang et al., 2000). In support of this, activating phosphorylation of SRC in *Trp53/Pten*^−/−^ cells increases ~3-fold with development of ispinesib resistance (Figure 3B), and treatment of four ispinesib-naive human GBM cell lines with 50 nM ispinesib enhances phosphorylated SRC 3- to 4-fold over vehicle by 24 h of treatment (Figure 3C). Since SRC phosphorylation can increase in fibroblasts during G_2_M (Chackalaparampil and Shalloway, 1988), a resistance-associated increase in SRC phosphorylation could simply reflect the effect of a G_2_M block. To test this, we treated ispinesib-naive GBM cells with nocodazole for 24 h to produce a G_2_M block and found that this treatment does not alter SRC phosphorylation (Figure S2).

### Ispinesib resistance can be reversed by simultaneously targeting EGFR and SFKs

While inhibiting EGFR by itself restores sensitivity to Kif11 inhibitors in other cell systems (Mardin et al., 2013), this is not the case in GBM (Figure 2D). One explanation is that while EGFR activity may be necessary for resistance, it is insufficient by itself and requires contributions from SFKs as well. This would predict that combining the EGFR inhibitor erlotinib with the SFK inhibitor dasatinib should synergize. Drugs can synergize in efficacy, in potency, or in both. Synergistic efficacy measures the percentage increase in the maximal effect of the two drugs compared with either drug alone, while synergistic potency measures how the presence of one drug changes the EC_50_ of the other. To deconvolve these two forms of synergy, we used our recently developed MuSyC algorithm (Wooten et al., 2021; Meyer et al., 2019). MuSyC fits a dose-response surface (Figure 3D, left) to drug-combination data to calculate the degree of synergistic efficacy (β) and synergistic potency (log(α12) and log(α21)). We monitored the change in cell count of ispinesib-resistant *Trp53/Pten*^−/−^ cells in the presence of 75 nM ispinesib as a function of time and across 191 different combinations of erlotinib and dasatinib. Cell growth was calculated by fitting the data to a series of exponential growth curves to generate rate constants for cell growth. We find that the combination of dasatinib and erlotinib is strongly synergistic in both efficacy and potency. Combining both drugs reduces the growth rate of ispinesib-resistant cells by up to 99% compared with either drug alone (Figure 3D, right; β_obs_ = 0.99). Importantly, while combinations of dasatinib and erlotinib produce a negative growth rate (reduction in cell count over time), neither drug alone can do this (Figure 3D, left, magenta shading). This conclusion does not depend on how we measure the effect of drug, as we also observe synergistic efficacy when we use static cell count at 96 h post treatment with drugs (Figure S3A). Furthermore, the presence of dasatinib increases the potency of erlotinib, reducing the concentration of erlotinib required to reach the EC_50_ by over 1,200-fold (log(α12) = 3.09) (Figure 3D, right, solid versus open circle). Erlotinib also increases the potency of dasatinib by nearly 15-fold (log(α21) = 1.17).

These findings would predict that saracatinib, a drug that inhibits both SFKs and EGFR (Hennequin et al., 2006), should sensitize resistant cells to ispinesib. We tested this prediction by treating ispinesib-resistant *Trp53/Pten*^−/−^ cells with combinations of saracatinib and ispinesib and performing MuSyc analysis as above. The combination of saracatinib + ispinesib is synergistic in both potency and efficacy (Figure 3E). Saracatinib sensitizes these cells to lower doses of ispinesib (log(α12) = 2.38; Figure 3E, red lines) while simultaneously increasing the maximal observed efficacy (Figure 3E; β_obs_ = 0.95). We likewise observe very similar findings when we monitor effects by static cell count at 96 h (Figure S3B). Taken together, these results suggest that EGFR and SFKs synergize in activating downstream effector(s) to reverse ispinesib resistance. We also complemented our Mu-Syc analysis with highest single agent (HSA) analysis for the same combinations of dasatinib + erlotinib, using cell growth rates (Figure 3F) and static cell counts (Figure 3G) as readouts. HSA measures synergy at each dose pair, in contrast to MuSyc, which classifies synergy based on the entire dose-response surface (Wooten et al., 2021). Nevertheless, we find that over nearly the entire range of combinations, the HSA synergy score is > 0 (red), implying synergy.

### STAT3 is a downstream effector of both EGFR and SFK, and its inhibition reverses ispinesib resistance in murine and human GBM

**Figure 4.**
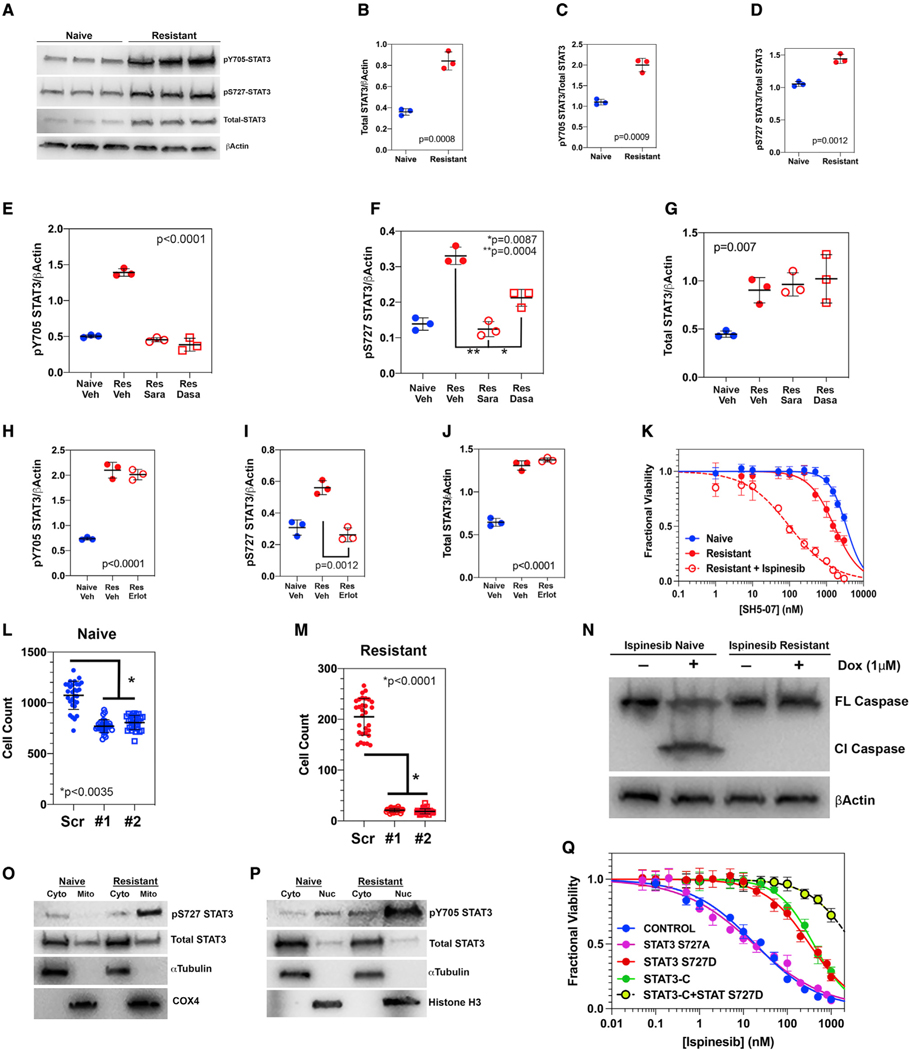
Ispinesib resistance depends upon STAT3 in murine *Trp53/Pten*^−/−^ GBM cells (A) Immunoblots for pY705 STAT3, pS727 STAT3, and total STAT3 in *Trp53/Pten*^−/−^ cells that are ispinesib naive and resistant. (B–D) Quantitation of total STAT3/β actin (B), pY705 STAT3/total STAT3 (C), and pS727 STAT3/total STAT3 (D) in ispinesib-naive (blue) and -resistant (red) cells, with three biological replicates of each. Statistical significance assessed with a two-tailed t test. (E and F) Western blot intensity, normalized to β actin, of pY705 STAT3 (E) and pS727 STAT3 (F) in ispinesib-naive (Naive) and -resistant (Res) cells, with the former treated with DMSO vehicle (Veh) and the latter treated with DMSO vehicle (Veh), 500 nM saracatinib (Sara), or 500 nM dasatinib (Dasa). (G) Western blot intensity, normalized to β actin, of total STAT3 in ispinesib-naive (Naive) and -resistant (Res) cells, with the former treated with DMSO vehicle (Veh) and the latter treated with DMSO vehicle (Veh), 500 nM saracatinib (Sara), or 500 nM dasatinib (Dasa). For (E)–(G), please refer to Figures S5A and S5B for the corresponding images of the western blots. Statistical significance determined using a pairwise two-tailed t test. (H–J) Quantitation of pY705 STAT3 (H), pS727 STAT3 (I), and total STAT3 (J), all normalized to β actin for ispinesib-naive cells treated with vehicle (solid blue circles; Naive Veh), ispinesib-resistant cells treated with vehicle (solid red circles; Res Veh), and ispinesib-resistant cells treated with 500 nM erlotinib (open red circles; Res Erlot). Statistical significance was determined by pairwise two-tailed t test. For (H)–(J), please refer to Figure S5C for the corresponding images of the western blots. (K) Dose-response curves for ispinesib-naive (solid blue circles) and -resistant (solid red circles) cells in the absence and presence of 75 nM ispinesib (open red circles) for the STAT3 inhibitor SH5–07. (L and M) Effect of shRNA suppression of STAT3 on ispinesib resistance. Please refer to Figure S5F for the corresponding western blot for STAT3, which shows >90% knockdown with two STAT3-directed shRNAs. Statistical significance determined using a pairwise two-tailed t test. (N) Ispinesib-naive and -resistant cells were treated for 24 h with 1 μM doxorubicin, and cell lysates were probed by western blot for full-length caspase 3 (FL Caspase 3) and cleaved caspase 3 (Cl Caspase 3). (O) Ispinesib-naive and -resistant cells were lysed, and mitochondria (Mito) were separated from the cytoplasm (Cyto) by centrifugation. Western blot shows that ispinesib resistance is associated with a marked increase in mitochondrial STAT3 that is phosphorylated on S727. Loading controls include α tubulin for the cytoplasmic and cytochrome c oxidase (*COX4*) for the mitochondrial fractions. (P) Ispinesib-naive and -resistant cells were lysed, and nuclei (Nuc) were separated from the cytoplasm (Cyto) by centrifugation. Western blot shows that ispinesib resistance is associated with a marked increase in nuclear STAT3 that is phosphorylated on Y705. Loading controls include a tubulin for the cytoplasmic and histone H3 for the nuclear fractions. (Q) Ispinesib-naive cells were transfected with STAT3 S727A, STAT3 S727D, STAT3-C, or STAT3 S727D + STAT3-C constructs, each fused to the FLAG epitope. After confirmation of expression of the STAT3 mutant (Figure S5G), cells were treated with a range of ispinesib concentrations, and cell viability was measured at 72 h. While transfection of either STAT3-S727D or STAT3-C increases the EC_50_ of ispinesib by ~20-fold, co-transfection of both of these constructs is required to increase the ispinesib EC_50_ to that seen for ispinesib-resistant cells (Figure 1A; Table S1).

The transcription factor STAT3 is a driver of the mesenchymal phenotype in GBM (Carro et al., 2010) and, through its anti-apoptotic effects, can also drive resistance to alkylating and targeted therapies (Fan et al., 2020; Tan et al., 2019; Zhao et al., 2016). We found upregulation of two STAT3-relevant pathways in ispinesib-resistant *Trp53/Pten*^−/−^ cells: the IL-6-JAK-STAT3 and interferon alpha response gene signatures (Figure 2B). STAT3 is activated primarily by phosphorylation at two sites: Y705, through the actions of either SRC or JAK kinases, and S727, through the actions of kinases downstream of EGFR (Aznar et al., 2001; Decker and Kovarik, 2000; Zhang et al., 2000). Furthermore, activation of STAT3 enhances its own expression (Narimatsu et al., 2001). We find that total STAT3 (normalized to β actin) increases 2- to 3-fold in resistant cells (Figures 4C, 4D, S5, and S5B); the fraction of STAT3 phosphorylated at Y705 and S727 increases 1.5- to 2-fold (Figures 4C, 4D, S5, and S5B); the amount of pY705 STAT3 and pS727 STAT3 normalized to β actin increases 2- to 3-fold (Figures 4E, 4F, S5A, and S5B); and phosphorylation of AKT is increased with ispinesib resistance (Figure S5D), consistent with the increased expression and activation of EGFR. Treatment of ispinesib-resistant cells with 500 nM saracatinib reduces levels of pY705 STAT3, pS727 STAT3, and pAKT to those of drug-naive cells (Figures 4E, 4F, S5A, S5B, and S5D) while not affecting total STAT3 levels (Figure 4G). While 500 nM dasatinib also reduces pY705 STAT3 levels to those of drug-naive cells (Figure 4E), it is significantly less effective in reducing pS727 STAT3 or pAKT levels (Figures 4F, S5A, S5B, and S5D). Conversely, while the EGFR inhibitor erlotinib reduces pS727 STAT3 levels to those of drug-naive cells (Figures 4I and S5C), it has no effect on levels of pY705 STAT3 (Figures 4H and S5C), on total STAT3 levels (Figures 4J and S5C), or on SRC phosphorylation (Figure S5E).

These results suggest that activated STAT3 mediates resistance to ispinesib in GBM. To test this, we examined the dose response of our *Trp53/Pten*^−/−^ ispinesib-naive and -resistant cells to the STAT3 inhibitor SH5–07 (Yie et al., 2016). Both naive and resistant cells are relatively resistant to SH5–07 in the absence of ispinesib. However, 75 nM ispinesib, a concentration that is toxic to ispinesib-sensitive but not to ispinesib-resistant cells (Figure 1A), shifts the SH5–07 dose-response curve >16-fold to the left (Figure 4K). We also suppressed STAT3 in our murine *Trp53/Pten*^−/−^ ispinesib-resistant and -naive GBM cells with short hairpin RNA (shRNA) (Figure S5F) and counted cells after 5 days of transfection. STAT3 suppression in ispinesib-naive cells reduces the cell count by 14%–21% (p < 0.035, two-tailed t test; Figure 4L), while for ispinesib-resistant cells in the presence of 75 nM ispinesib, it reduces the cell count by >5-fold (p < 0.0001, two-tailed t test; Figure 4M). Phosphorylation of STAT3 on Y705 translocates it to the nucleus, where it activates transcription of a variety of genes that promote oncogenesis, including those inhibiting apoptosis (Diallo and Herrera, 2021). Phosphorylation of STAT3 on S727 induces it to localize in the mitochondria, where it has multiple downstream effects, including inhibition of apoptosis. Our proposal that increases in pY705 and pS727 STAT3 lead to apoptosis resistance is supported by the western blot in Figure 4N. We treated ispinesib-naive cells with 1 μM doxorubicin for 24 h, and this induced the appearance of cleaved caspase 3 (Figure 4N, left). However, doxorubicin has no such effect in ispinesib-resistant cells (Figure 4N, right).

By increasing STAT3 phosphorylation at S727 and Y705, ispinesib resistance should therefore lead to mitochondrial accumulation of the former and nuclear accumulation of the latter. We therefore measured pS727 STAT3 in cytoplasmic and mitochondrial fractions (*Cyto* and *Mito*, respectively; Figure 4O) and pY705 in cytoplasmic and nuclear fractions (*Cyto* and *Nuc*, respectively; Figure 4P) of ispinesib-naive and -resistant cells. Development of ispinesib resistance increases the mitochondrial content of pS727 STAT3 and the nuclear content of pY705 STAT3. To test the functional significance of these post-translational modifications on ispinesib resistance, we transfected ispinesib-naive *Trp53/Pten*^−/−^ cells with plasmids encoding for one of three mutant STAT3 constructs: a non-phosphorylatable S727A, a phosphomimetic S727D construct, and an A662C, N664C double mutant (referred to as STAT3-C) that mimics the effect of Y705 phosphorylation (Bromberg et al., 1999), with each mutant fused to the FLAG epitope. Western blots of transfected cell lysates demonstrate expression of this epitope at the expected molecular weight (Figure S5G). While expression of the S727A STAT3 mutant has essentially no effect on ispinesib EC_50_, expression of either the S727D STAT3 or the STAT3-C phosphomimetic increases the EC_50_ approximately 20- to 25-fold, implying that these two post-translational modifications provide a similar but very limited degree of resistance to ispinesib (Figure 4Q; Table S1). However, when we co-transfect *Trp53/Pten*^−/−^ cells with both the S727D and STAT3-C encoding plasmids, the EC_50_ for ispinesib shifts >180-fold to the right, to a value that is very similar to that for ispinesib-resistant cells (Figure 1A; Table S1).

While ispinesib resistance is not associated with upregulation of Kif15, it may still be possible that Kif15 plays a role in the biology of resistance. To test this, we treated naive and resistant *Trp53/Pten*^−/−^ GBM cells with the Kif15 inhibitor Kif15-IN-1 and allowed cell proliferation to continue for 72 h before measuring viable cell content. Neither naive nor resistant cells in the absence of ispinesib are sensitive to Kif15-IN-1 (Figure S4). However, in the presence of 75 nM ispinesib, resistant cells could be fit to a dose-response curve that assumes a maximum reduction in cell content of 45%, associated with an EC_50_ (456 ± 52 nM; Figure S4) that is in the range of what has been reported for this drug (Table S1; Dumas et al., 2019; Milic et al., 2018). These results imply that in the absence of combined Kif11 or Kif15 function, ispinesib-resistant GBM cells stop proliferating but they remain viable, reflecting the defects in apoptosis generated by STAT3 activation.

**Figure 5.**
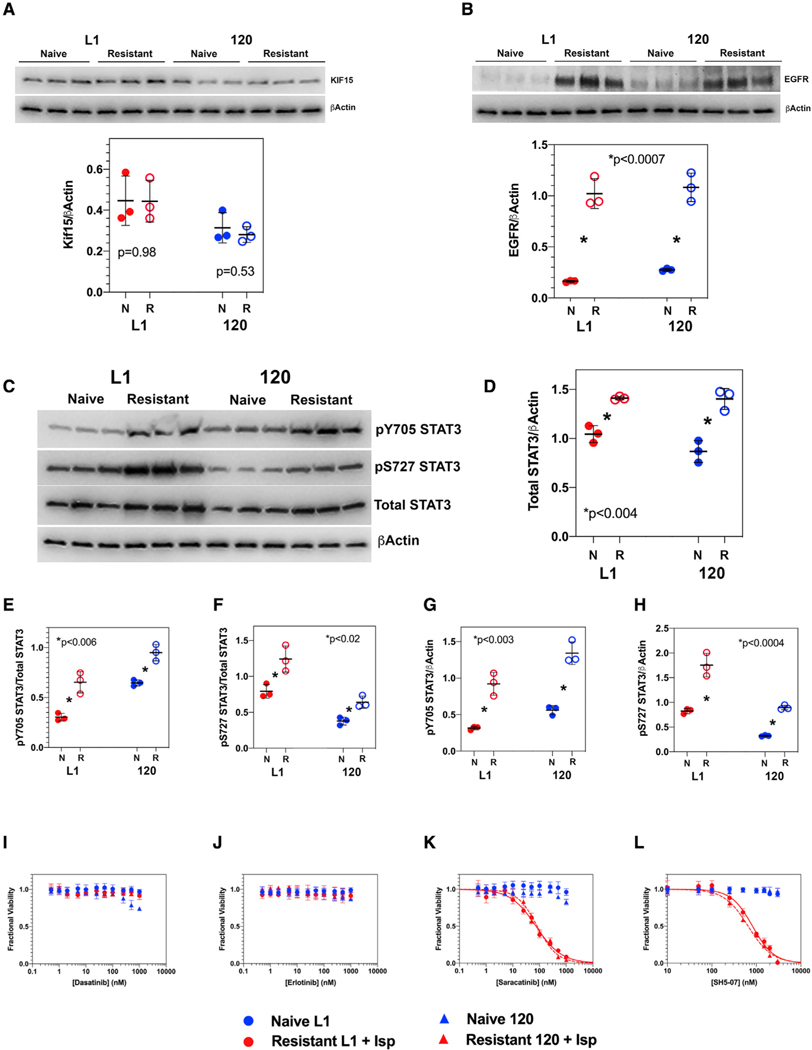
Ispinesib resistance depends upon STAT3 in human GBM (A) Western blots of two human GBM lines (L1 and 120) show no significant upregulation of Kif15 between ispinesib-naive (*N*) and -resistant (*R*) lines. Significance determined by a two-tailed t test. (B) Expression of EGFR is increased 3- to 4-fold in resistant (*R*) L1 and 120 GBM lines compared with the corresponding drug-naive (*N*) lines. Statistical significance determined using a pairwise two-tailed t test. (C–H) Phosphorylation of STAT3 increases in the human GBM cell lines L1 and 120 with development of ispinesib resistance. (C) Western blot for pY705, pS727, and total STAT3 in ispinesib naive (*N*) and resistant (*R*) GBM L1 and 120 cell lines. (D–H) Quantitation of total STAT3/β actin (D), pY705 STAT3/total STAT3 (E), pS727 STAT3/total STAT3 (F), pY705 STAT3/β actin (G), and pS727 STAT3/β actin (H) in ispinesib-naive (*N*) and -resistant (*R*) L1 and 120 cell lines. Statistical significance was determined by a two-tailed t test. Statistical significance determined using a pairwise two-tailed t test. (I–L) Ispinesib resistance in human L1 and 120 GBM cell lines can be reversed with saracatinib and SH5–07 but not with either erlotinib or dasatinib. (I and J) Dose-response curves for dasatinib (I) and erlotinib (J) show no effect of either drug as a single agent on ispinesib-naive (blue) or -resistant (red) cell lines, the latter in the presence of 50 nM ispinesib. (J and K) By contrast, both saracatinib (K) and SH5–07 (L) are active against ispinesib-resistant L1 and 120 cells in the presence of 50 nM ispinesib (red curves) but not against ispinesib-naive (blue curves) L1 and 120 cells. Relevant EC_50_ values are listed in Table S1.

To extend these observations to human GBM, we generated ispinesib-resistant versions of two primary human GBM lines (L1 and 120). Immunoblots of ispinesib-naive (Figures 5A–5H) and -resistant (Figures 5A–5H) L1 and 120 GBM cells reveal that acquisition of resistance does not increase Kif15 expression but does increase expression of EGFR (Figures 5A and 5B). When normalized to β actin, the total levels of STAT3, pY705 STAT3, and pS727 STAT3 are all increased as is the fraction of total STAT3 that is phosphorylated at both Y705 and S727 (Figures 5C–5H). We performed dose-response studies of single agent erlotinib, dasatinib, saracatinib, and SH5–07 by incubating these cells with these drugs in the presence of 50 nM ispinesib and measuring cell viability (Figures 5I–5L). Consistent with our murine *Trp53/Pten*^−/−^ model, ispinesib resistance in these human GBM lines can be reversed with saracatinib and SH5–07 but not by single agent erlotinib or dasatinib. We used MuSyC analysis to generate a dose-response surface for the effects of combining erlotinib and dasatinib, and this demonstrates a marked degree of synergy of efficacy (β_obs_ = 5.09) corresponding to a >500% reduction in cell growth at the maximum test dose of both drugs (Figure S3C). Since neither erlotinib nor dasatinib alone have any appreciable effect on ispinesib-resistant cells in the presence of 50 nM ispinesib (Figures 5I, 5J, and S3C), we were not able to get reliable measures of EC_50_s for these drugs as single agents, precluding our ability to measure synergistic potency.

### Single-cell RNA-seq demonstrates that ispinesib-resistant *Trp53/Pten*^−/−^ cells co-express elevated levels of SRC and EGFR and activate STAT3 signaling

**Figure 6.**
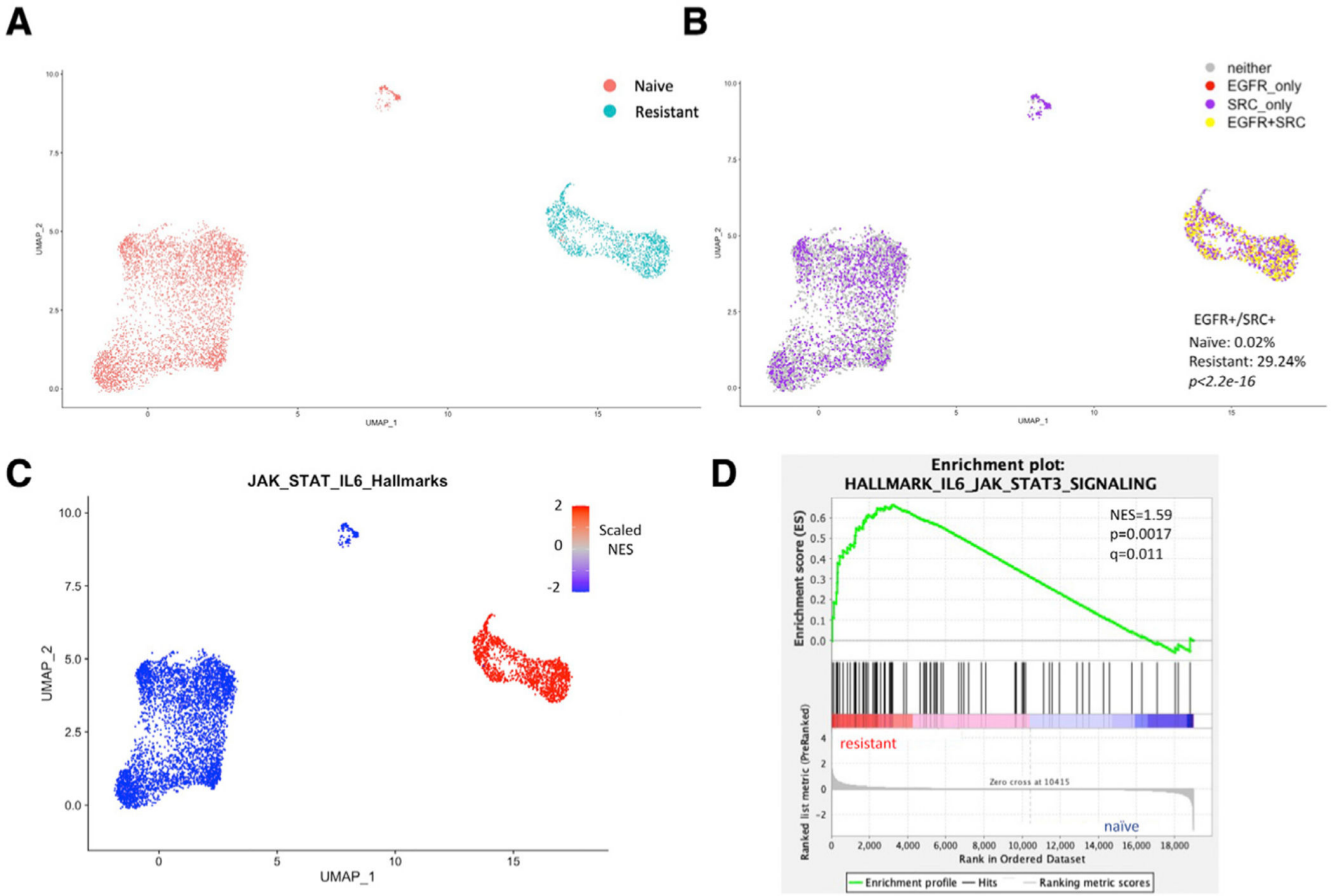
scRNA-seq analysis of naive and ispinesib-resistant MGPP3 murine cell lines (A) Uniform manifold approximation and projection (UMAP) of all cells in the dataset, annotated by whether they are part of the naive or resistant populations. (B) Co-expression analysis of EGFR and SRC transcripts on original dataset. Cells were deemed EGFR or SRC positive based on whether they express any transcripts of the respective gene. See Figure S6B for subsample analysis to equalize coverage across conditions before doing co-expression analysis. (C) Cluster-by-cluster gene set enrichment analysis (GSEA) of the Cancer Hallmarks ‘‘IL6_JAK_STAT3_SIGNALING’’ pathway. Each cluster is colored based on its normalized enrichment score (NES) for the pathway. (D) GSEA of same IL-6/JAK/STAT3 pathway comparing all resistant cells with all naive cells. Normalized enrichment score, as well as p value and false discovery rate (FDR) q value, are displayed.

Single-cell RNA-seq (scRNA-seq) was performed on both ispinesib-naive and -resistant *Trp53/Pten*^−/−^ cells (Figure 6A). Cluster analysis (Levine et al., 2015) revealed 7 clusters of cells, 5 of which are associated with ispinesib-naive cells and 2 with ispinesib-resistant cells (Figure S6A). Differential gene expression analysis demonstrates that the transcripts for *Egfr* (p < 10^−300^) and *Src* (p *=* 2 X 10^−238^) are significantly enriched in the resistant sample (Figures S6C and S6D). Notably, transcripts of *Egfr* were detected in 36% of cells in the resistant population compared with only 0.1% in the naive population. Cells were characterized as *Src* positive, *Egfr* positive, both, or neither based on whether they express any transcripts for either *Src* or *Egfr*. Importantly, 29.2% of the resistant cells were positive for both (double positive), whereas only 0.1% of drug-naive tumor cells were double positive (Figure 6B). After subsampling of transcript counts to normalize coverage across conditions, *Egfr* and *Src* transcripts were significantly co-expressed in the resistant population compared with the naive population (p < 2 × 10^−16^; Figure S6B). In resistant cells, the transcript for *Stat3* was also detected at a significantly higher rate compared with naive cells (p *=* 2 × 10^−5^; Figure S6E), with *Stat3* detected in 88% of resistant cells compared with 62% in the drug-naive group. Cluster-specific gene set enrichment analysis (GSEA) revealed that resistant clusters are significantly enriched in the IL-6/JAK/STAT3 pathway (Figure 6C), a finding recapitulated when comparing all resistant cells with all naive cells via GSEA (Figure 6D).

### Saracatinib enhances ispinesib efficacy in murine models of GBM

**Figure 7.**
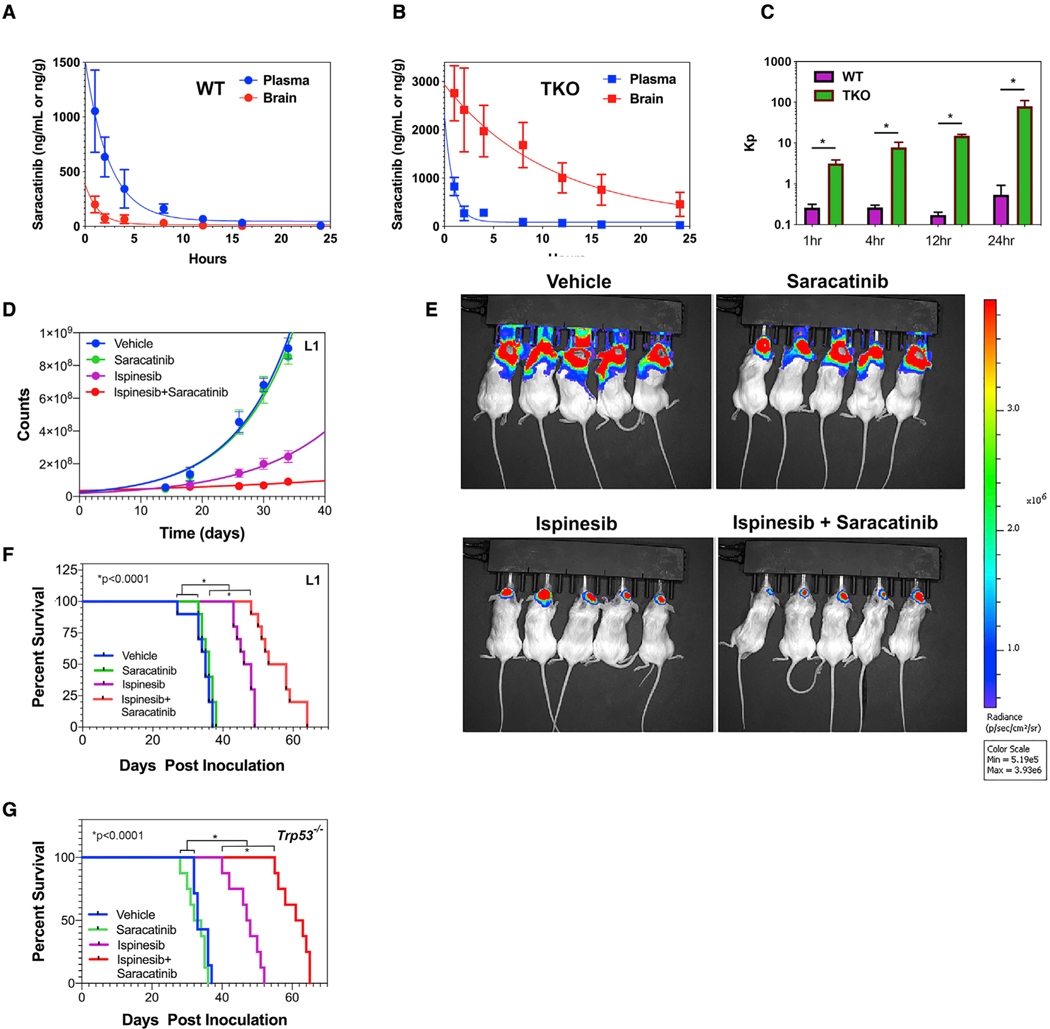
Saracatinib enhances the efficacy of ispinesib in murine and human GBM pre-clinical models (A and B) Plot of saracatinib concentrations in plasma (blue) and brain (red) versus time after intravenous (i.v.) administration of a single dose, given to wild-type (WT) FVB mice (A) and FVB mice with homozygous deletions of the ABCB1 and ABCG2 efflux transporters (TKO; B). Data for both plasma and brain levels versus time were fit to single exponential decays, yielding pharmacokinetic parameters summarized in Table S5. Loss of the ABCB1 and ABCG2 efflux transporters increases brain half-life of saracatinib 7.5-fold. (C) Values of K_p_, the brain-to-plasma concentration ratio of saracatinib, in WT and TKO mice versus time after injection. At each time interval, differences between WT and TKO mice are statistically significant (p < 0.04, two-tailed t test). (D) Growth kinetics of a GBM PDX treated with vehicle (DMSO; blue), saracatinib (green), ispinesib (magenta), or ispinesib + saracatinib (red). NSG mice were orthotopically injected with 100,000 luciferase-expressing L1 human GBM cells, and luminescence was monitored over the subsequent 35 days. (E) Bioluminescence images corresponding to day 34 post-injection from (D). (F) Kaplan-Meier survival curves for NSG mice orthotopically injected with 100,000 luciferase-expressing L1 cells and subsequently treated with vehicle (DMSO; blue), saracatinib (green), ispinesib (magenta), or ispinesib + saracatinib (red). Differences in survival between saracatinib + ispinesib and ispinesib treatment and between ispinesib and vehicle or saracatinib treatment are highly significant (p < 0.0001, log rank test). (G) Corresponding Kaplan-Meier curves for mice with orthotopic *Trp53*^−/−^ GBMs. As with (F), the combination of ispinesib + saracatinib is appreciably superior to ispinesib treatment alone (p < 0.0001, log rank test).

We administered a single dose of saracatinib to 28 mice and determined its plasma and brain concentrations over the subsequent 24 h using liquid chromatography with tandem mass spectrometry (LC-MS/MS). We used two strains of mice–wild-type FVB and FVB mice with genetic deletion of the two major brain efflux transporters (*Abcb1 and Abcg2*), referred to as triple knockout (TKO) mice. Plasma and brain drug concentrations versus time are illustrated in Figures 7A for wild-type and 7B for TKO mice, and data were fit to single exponential decays. Figure 7C depicts the value of K_p_, the brain-to-plasma concentration ratios of saracatinib, in wild-type and TKO mice versus time after injection. These data allowed us to calculate values of elimination half-life (t_1/2_), clearance (Cl), and total area under the concentration-time curve $\left({\text{AUC}}_{0\to \infty }\right)$ for saracatinib (Table S5). The brain concentrations of saracatinib in TKO mice are greater than those in wild-type mice for the duration of the time course, and consequently, the overall brain-to-plasma AUC ratio in TKO mice is also higher. This indicates that saracatinib is a substrate for efflux from the brain by either ABCB1 or ABCG2. Corresponding data for dasatinib have been previously published, and a comparison shows that while saracatinib is subject to efflux, the overall brain-to-plasma AUC ratio for saracatinib is double that of dasatinib (Chen et al., 2009). The brain exposure of dasatinib is less than 10% of the dose (Chen et al., 2009), while that for exposure of saracatinib is nearly 25%.

These results predict that saracatinib should prevent or retard the emergence of ispinesib resistance in an *in vivo* mouse model of GBM and thereby prolong survival over ispinesib treatment alone. We tested this in an orthotopic patient-derived xenograft (PDX) model using the L1 human GBM line that was transfected with a luciferase reporter. Ten mice per group were treated with vehicle, ispinesib, saracatinib, or saracatinib + ispinesib 14 days after orthotopic injection of 100,000 tumor cells. Tumor size was monitored by bioluminescence imaging over the subsequent 35 days (Figures 7D and 7E). Luminescence counts were fit to single exponential growth equations, yielding doubling times of 6.5 ± 0.6 days for vehicle, 6.6 ± 0.6 days for saracatinib, 9.1 ± 1.0 days for ispinesib, and 28.2 ± 7.0 days for ispinesib + saracatinib. As expected from these results, survival of these mice is markedly prolonged with combination therapy compared with single agent ispinesib or saracatinib (Figure 7F), while treatment with saracatinib alone has no significant effect. The superiority of combination therapy is also evident in our *Trp53*^−/−^ GEMMs (Figure 7G).

## DISCUSSION

### While the initial clinical experience of mitotic kinesin inhibitors has been disappointing, more recent developments have renewed interest in these drugs

The importance of microtubules in driving mitosis motivated the development of a variety of microtubule toxins as anti-cancer therapeutics. While these drugs have met with success in treating hematologic and solid malignancies (Komlodi-Pasztor et al., 2012; Perez, 2009), their neurotoxicity is problematic. By contrast, the mitotic kinesins have been an appealing alternative target since they also drive mitosis, but their inhibition is not neurotoxic. This has led to development of >50 small-molecule inhibitors of one of these, Kif11, which is involved in formation of the bipolar mitotic spindle. Although phase I and II trials have demonstrated acceptable toxicity of these inhibitors, their clinical efficacy has been disappointing (Chandrasekaran et al., 2015; Rosenfeld, 2015), reflecting drug resistance. Nevertheless, several developments have renewed interest in mitotic kinesin inhibitors, particularly for CNS malignancies. These include the observations that mitotic kinesins are upregulated in aggressive meningioma and in malignant peripheral nerve sheath tumors (Nassiri et al., 2021; Terribas et al., 2020), that Kif11 inhibitors are active against meningioma (Jungwirth et al., 2021), and that upregulation of another mitotic kinesin, Kif20A, is associated with worse outcomes in male patients with GBM (Yang et al., 2019). We had previously shown that ispinesib is CNS permeant and significantly prolongs survival in murine PDX models of GBM (Venere et al., 2015; Gampa et al., 2020). We now find that resistance to ispinesib in GBM occurs by a mechanism distinct from those previously described for this drug (Kasap et al., 2014; Sturgill and Ohi, 2013; Mardin et al., 2013; Gampa et al., 2020) and that targeting this mechanism of resistance significantly enhances ispinesib efficacy.

### Resistance to ispinesib in GBM is driven by STAT3

The cell enlargement and polyploidy in ispinesib-resistant GBM cells suggests that the mechanism driving resistance works by suppressing the mitotic catastrophe and apoptotic cell death that ordinarily follow a G_2_M arrest (Vakifahmetoglu et al., 2008). Ispinesib resistance is associated with upregulation of both apoptotic and inflammatory pathways, and STAT3 connects to both, as well as to the proneural-to-mesenchymal transition that accompanies ispinesib resistance. Along with CEBPB, STAT3 is a master transcriptional regulator of the mesenchymal phenotype in GBM (Carro, MS et al., 2010). Through inhibiting apoptosis and driving self-renewal, STAT3 also mediates resistance to both chemo- and targeted therapeutics (Fan et al., 2020; Tan et al., 2019; Zhao et al., 2016; Sherry et al., 2009). While GBMs are typically resistant to STAT3 inhibition (Fan et al., 2013, 2020; Miyata et al., 2017), both SH5–07 and STAT3 shRNA are specifically toxic to resistant GBM cells in the presence of ispinesib, consistent with the uniform enhancement of IL6-JAK-STAT3 signaling that we observe solely among resistant cells.

### Phosphorylation-dependent regulation connects STAT3 to SRC, EGFR, and ispinesib resistance

Phosphorylation of STAT3 at Y705 by SRC and JAK leads to transcription of genes involved in cancer progression, including those regulating apoptosis (Diallo and Herrera, 2021). Furthermore, Y705 phosphorylation is enhanced by the physical association of STAT3 with PDGFRβ (Wang et al., 2000; Vignais and Gilman, 1999), which is upregulated nearly 3-fold in resistant cells. Phosphorylation of STAT3 at S727 localizes it to the mitochondria, where it closes the mitochondrial permeability transition pore and blocks the cytoplasmic release of cytochrome c that normally occurs in response to pro-apoptotic stimuli (Wegrzyn et al., 2009; Gough et al., 2009; Decker and Kovarik, 2000; Diallo and Herrera, 2021). Thus, phosphorylation of STAT3 at these two sites has complementary anti-apoptotic effects, which provides a structural basis for the synergy between SRC and EGFR inhibitors in reversing ispinesib resistance. This synergy does not imply that individual STAT3 molecules need to be phosphorylated at both sites. Rather, we propose that the fully resistant phenotype requires sufficient concentrations of pY705 STAT3 and of pS727 STAT3 molecules to suppress both the nuclear and the mitochondrial contributions to apoptosis, respectively. Our finding that co-transfection of both S727D STAT3 and STAT3-C is sufficient to completely induce ispinesib resistance supports this proposal.

The importance of both SRC and EGFR to ispinesib resistance in GBM is supported by several findings in our study. First, cells that upregulate both are only found in the ispinesib-resistant group. While dasatinib inhibits SFKs with subnanomolar EC_50_, it also can inhibit MEK with a much lower potency (EC_50_ = 1,700 nM; Lombardo et al., 2004), and given the concentrations used in this study (500 nM), this feature could explain why dasatinib can partially reduce pS727 STAT3 levels. Thus, in the presence of the highly specific EGFR inhibitor erlotinib, less dasatinib would be needed to produce half maximal cell kill. Likewise, combined targeting of both SFKs and EGFR with saracatinib would reduce the concentration of ispinesib needed for cytotoxicity in resistant cells. Conversely, overcoming resistance completely should require preventing phosphorylation at both sites, which could explain why the combination of dasatinib and erlotinib also demonstrates synergy of efficacy. Furthermore, if upregulation of either SFKs or EGFR can generate some resistance to ispinesib, we might also expect heterogeneity among resistant cells, with some upregulating one, the other, or both kinases, and this prediction is consistent with our scRNA-seq findings.

### For a targeted drug to be effective in GBM, it may need to be combined with a strategy that inhibits the development of resistance

Our work demonstrates how a promising therapeutic, which failed as a single agent in numerous clinical trials and was abandoned, can in fact be rendered effective by anticipating and prospectively treating *ab initio* the mechanism that drives treatment resistance. Since EGFR amplification or activating mutations are present in up to 57% of GBMs (An et al., 2018), and since SRC phosphorylation can be detected as early as 6 h after exposure of GBM cells to ispinesib, development of STAT3-mediated resistance could develop rapidly. In support of this, our Kaplan-Meier survival studies in a *Trp53*^−/−^ GEMM and a human PDX model both show that while saracatinib by itself has no effect on survival, it significantly improves the effectiveness of ispinesib, presumably by retarding development of ispinesib resistance.

Our results with Kif15-IN-1 imply that when ispinesib-resistant GBM cells are deprived of Kif11 function, they turn to Kif15 to continue to proliferate. While this might argue that combining ispinesib with a Kif15 inhibitor might also prevent development of ispinesib resistance, we note that Kif15-IN-1 is effective only in the presence of ispinesib (Figure S4). Since ispinesib and Kif15-IN-1 both inhibit spindle function in mitosis, they likely have overlapping dose-limiting toxicities, and combining these two drugs together would likely be highly toxic. By contrast, inhibitors of STAT3 work by a mechanism distinct from ispinesib—by enabling tumor cells to undergo the mitotic catastrophe that ordinarily follows a G_2_M block. This likely explains why combinations of ispinesib and saracatinib can be administered to both GEMMs and PDX murine models at doses that are both effective and well tolerated. Combining a drug that blocks a driving oncogene with another drug that is devoid of overlapping toxicities and that blocks a compensatory signaling pathway is already clinically employed, as exemplified by the combination of RAF and MEK inhibitors in the treatment of a variety of malignancies (Yaeger and Corcoran, 2019). While our studies demonstrate that phosphorylation-dependent activation of STAT3 can drive ispinesib resistance, future studies will be needed to determine how widespread this mechanism is across the spectrum of tumors that constitute GBMs.

### Limitations of the study

Our work has established that induction of apoptosis resistance by STAT3 is the driving mechanism behind ispinesib resistance in GBM. However, our results are not inconsistent with the possibility that previously established mechanisms may also contribute to the resistant phenotype, and we have not explored whether targeting any of these mechanisms synergizes with a STAT3-inhibitory strategy. This issue is relevant to clinical applications of our findings since, for example, efflux transporters can be inhibited with clinically available drugs. A second limitation is that the *in vitro* studies described in this report do not recapitulate the cellular or mechanical features of the microenvironments that characterize a GBM. Although predictions made from these experiments are consistent with *in vivo* studies of combinations of ispinesib and saracatinib in murine GBM models, it remains possible that local variations in microenvironment from tumor to tumor may alter the efficacy of combined Kif11 and STAT3 targeting.

## STAR★METHODS

### RESOURCE AVAILABILITY

#### Lead contact

Further information and requests for resources and reagents should be directed to and will be fulfilled by the lead contact, Steven S. Rosenfeld (rosenfeld.steven@mayo.edu).

#### Materials availability

Genetically engineered mouse models and cell lines will be distributed after completion of the relevant Materials Transfer Agreements with the Mayo Clinic.

#### Data and code availability

- Bulk and single cell RNA-seq data have been deposited in the Gene Expression Omnibus (GEO) database, with accession numberGEO: GSE193180, and with sub series dataset accession numbersGEO: GSE193178 (bulk RNA-seq) and GEO: GSE193179 (single cell RNA-seq).
- The mass spectrometry proteomics data have been deposited into the ProteomeXchange Consortium via the PRIDE (Perez-Riverol et al., 2019) partner repository with the dataset identifier PRIDE: PXD030715 and https://doi.org/10.6019/PXD030715.
- Accession information is available in the Key resources table. All other data reported in this paper will be shared by the lead contact upon request.
- This paper does not report any original code.
- Any additional information required to reanalyze the data is available from the lead contact upon request.

### EXPERIMENTAL MODEL AND SUBJECT DETAIL

#### Mice

All mouse procedures were performed in compliance with the Mayo Clinic Institutional Animal Care and Use Committee guidelines. Homozygous floxed *Trp53* mice (Stock #008462) and NOD-SCID mice (Stock #005557) were obtained from Jackson Laboratory. Studies were performed on equal numbers of male and female mice, ages 8–20 weeks. Wild type FVB and TKO FVB mice were obtained from colonies housed at the Academic Health Center of the University of Minnesota, with original breeder pairs obtained from Taconic Biosciences, Inc. (Germantown, NY). Animal genotypes were regularly verified via tail snip (TransnetYX, Cordova, TN).

### METHOD DETAILS

#### Glioma cell line isolation from mouse GBM tumor and culture

The protocol for isolation of tumor cells from *Trp53*(−/−) and *Trp53/Pten*(−/−) murine tumors has been described (Lei et al., 2011). The human L1 primary GBM cell line was cultured and maintained in DMEM + F12 media with 1% N2 supplement (Gibco), 20ng/mL of hEGF (Sigma-Aldrich) and 20ng/mL of hFGF (R&D systems). Human GBM cells 1A, 108, and 120 primary lines were cultured and maintained in DMEM + F12 media with 1% NeuroPlex supplement (Gemini), 20ng/mL of EGF and 20ng/mL of FGF. Ispinesib resistant *Trp53/Pten*(−/−) murine GBM cells were generated by culturing in the presence of 75 nM of ispinesib for three weeks, and then maintained at this ispinesib concentration. Ispinesib resistant human L1 and 120 GBM cells were generated by culturing in the presence of 25 nM ispinesib for first week and 50 nM for second and third week. These cells were maintained in the presence of 50 nM ispinesib.

#### Dose response curves/cell viability assays

5,000 cells/well were plated in 96-well plates were treated 48 h later with various doses of ispinesib, saracatinib, dasatinib, erlotinib, or SH5–07. For experiments involving MuSyc drug synergy analysis (Figures 3D and 3E) the plates were scanned and phase-contrast images (4 per well) were acquired every 12 h for 4 days using a Cytation 5 Cell Imaging Multi-Mode Reader (BioTek Instruments, Inc). Cell counts per well at each time point were quantified and cell count *versus* time was fit to a set of exponential growth equations to calculate growth rate constants for each condition (Prizm). For dose response experiment in Figures S3A and S3B, cells were treated with drug for 96 h, fixed with ice-cold 4% paraformaldehyde for 20 min at room temperature, stained with DAPI to visualize the nuclei, and DAPI positive cells were counted using the Cytation 5 automated plate reader (BioTek). For the dose response experiments in Figures 1A, 1B, 2D, 2E, 4K, 4Q, 5I, 5L, S3C, and S4, cells were treated with drug for 72–96 h and cell viability was measured using CellTiter-Glo (Promega, cat# G9242).

#### Retrovirus production and intracerebral injections

PDGF-IRES-cre retrovirus was generated and injected intracranially according to methods described previously (Lei et al., 2011; Kenchappa et al., 2020). For the pharmacologic studies mice were treated five days after retroviral injection with vehicle, saracatinib (*25 mg/kg by oral gavage, 5 days per week*), ispinesib (*10 mg/kg by intraperitoneal injection for every 4 days*), or ispinesib + saracatinib (*ispinesib: 10 mg/kg by intraperitoneal injection for every 4 days; saracatinib:* 25 mg/kg *by oral gavage, 5 days per week*) with 7 mice per treatment group. Treatment continued until tumor morbidity. For studies using the L1 GBM PDX model, NOD/SCID mice were orthotopically injected with 100,000 human L1 GBM cells (kindly provided by Dr. Justin Lathia, Cleveland Clinic), and treatment with vehicle, ispinesib, and/or saracatinib as described above began 14 days later, with 10 mice per group. Treatment was continued until tumor morbidity.

#### Bioluminescence studies

Intracranial tumor implantation was performed on 8 week old post-natal NOD-SCID mice obtained from Jackson laboratory (stock #005557). Under isoflurane-induced anesthesia, 100,000 luciferase expressing human L1 GBM cells were injected in 1μL of culture media at coordinates X = 1.5mm, Y = 1.5mm and Z = 2.5mm relative to the bregma. Tumor formation was monitored by intraperitoneal injection of 3 mg/kg body weight of D-luciferin (Xenolight D-Luciferin firefly potassium salt, PerkinElmer, catalog# 122799) in sterile phosphate buffered saline (PBS) followed by IVIS imaging. Once photon counts reached between 2.5 and 7.0 × 10^7^/sec, animals were divided into four groups with 10 mice per group and administered vehicle, saracatinib (*25 mg/kg by oral gavage, 5 days per week*), ispinesib (*10 mg/kg by intraperitoneal injection for every 4 days*) or ispinesib + saracatinib (*10 mg/kg by intraperitoneal injection for every 4 days;* 25 mg/kg *by oral gavage, 5 days per week, respectively)*. IVIS imaging was performed every 4–8 days and photon flux was calculated from the resulting signal.

#### Immunofluorescence microscopy

Drug naïve and ispinesib resistant GBM cells were grown on glass bottom chamber slides and fixed with ice-cold 4% paraformaldehyde for 20 min at room temperature. After washing with PBS three times, cells were permeabilized with 0.1% Triton X-100 PBS, and then washed three more times with PBS. Cells were stained for F-actin with rhodamine phalloidin (Cytoskeleton Inc, cat #PHDR1, diluted to 100nM final concentration) for 30 min. The slides were then mounted with cover slips using Vectashield with DAPI (#H-1200; Vector Laboratories), and cells were visualized and imaged using confocal microscopy (Zeiss).

#### Western blots

Cells were scraped and incubated in lysis buffer (50 mM Tris HCl at pH 7.40, 150 mM NaCl, 1 mM EDTA, 1.0% Nonidet P-40, and a mixture of protease and phosphatase inhibitors), on ice from 30 min. Debris was removed by centrifugation for 10 min at high speed at 4°C, and cleared lysates were run on SDS/PAGE and transferred to polyvinylidene difluoride membranes. Membranes were blocked in 5% non-fat dry milk in TBS +0.1% Tween 20 for 1 h at room temperature, incubated with primary antibody in blocking solution for overnight at 4°C, followed by secondary antibody for 1 h at room temperature, and developed using an enhanced chemiluminescence solution.

#### Lentiviral production and transduction

Knockdown of STAT3 in murine GBM cells was achieved via lentiviral infection with shRNA-encoding constructs. The lentiviral plasmid vector pLKO.1-puro based shRNA clones and control shRNA vector were purchased from Sigma-Aldrich (St Louis, MO, USA). The following constructs were used for murine GBM cells in these studies: Non Targeting control (SHC002); STAT3 sh-RNA [TRCN0000071454 (STAT3-shRNA-1), TRCN0000071456 (STAT3-shRNA-2). Each of the pLK0.1 targeting constructs were co-transfected with psPAX2 and pMD plasmids into HEK-293T cells via Lipofectamine 3000 transfection agent (Life Technologies, catalog # 11668027) in serum-free medium. After 8 h of transfection, the viral particle-containing medium was removed and replaced with fresh complete medium. Transfected cells were then grown in DMEM media containing 10% FBS for 48 h at 37°C, 5% CO2. Media containing virus was harvested and centrifuged for 10 min in a clinical specimen centrifuge and then filtered through a 0.45 μm filter. Lentiviral particles were concentrated using LentiX-Concentrator reagent (Takara Bio USA) and the viral titer was determined using a p24 ELISA kit (Clontech). Mouse glioma cells were infected by incubating with virus containing media (at 10 MOI of virus and 4μg/mL of polybrene (Sigma-Aldrich)) overnight. Cells were selected for positive shRNA infection using puromycin (0.5ug/mL) for seven days and maintained in 0.1ug/mL puromycin containing media, and then effect of STAT3 knockdown on cell viability was measured.

#### Apoptosis assays with doxorubicin

Drug naïve and ispinesib resistant mouse glioma cells were treated with vehicle or 1 μM of doxorubicin (Selleck, cat# S1208) for 24 h and lysates were subjected to caspase 3 and β-actin Western blotting.

#### Mitochondrial localization of STAT3

Mitochondria and cytosolic fractions from ispinesib naïve and resistant murine lines were isolated (Mitochondria Isolation Kit for Cultured Cells, Abcam, cat# 110170). Protein concentration was measured with a BCA assay (and fractions were stored at −80°C until further use. Samples were subjected to Western blotting using anti-pS727-STAT3, anti-STAT3, anti-cytochrome oxidase type IV, and anti α-tubulin antibodies.

#### Preparation of S727A-STAT3 and S727D-STAT3 lentiviruses and transfection

STAT3 sequences from adenoviral plasmids encoding Flag-tagged mouse S727A-STAT3 (Addgene, #99262) and S727D-STAT3 (Addgene, #99263) were amplified by PCR (CloneAmp^™^ HiFi PCR, Takara Bio) using specific primers (CATTGGTAACTGTCAGAC CAAGTTTACTCA; TGAGCCATGGTGGCGCTAGCT; CGCCACCATGGCTCAGTGG; AGGCCGCTCTACTTGTCATCGTCA; CAAGT AGAGCGGCCTCGAGCATGC; GGTCTGACAGTTACCAATGCTTAATCAG for S727A-STAT3 and CATTGGTAACTGTCAGACCAA GTTTACTCA; TGAGCCATGGTGGCGCTAGCT; CGCCACCATGGCTCAGTGG; AGGCCGCTCTACTTGTCATCGTCA; CAAGTAGAG CGGCCTCGAGCATGC; GGTCTGACAGTTACCAATGCTTAATCAG for S727D-STAT3), and subcloned into the pLenti Lifeact-EGFP BlastR plasmid (Addgene, #84383) to generate S727A and S727D-STAT3 expressing lentiviral plasmids. The lentiviral plasmids were co-transfected with the plasmids psPAX2 and pMD.2 in HEK293T cells to produce lentiviral particles. As described above lentiviral particles were tittered and transduced (20 MOI of virus) into drug naïve mouse glioma cells and transduced cells were selected by Blasticidin treatment (7.5μg/mL, Sigma Aldrich) for 3 days. Then cells were treated with ispinesib and measured cell viability.

#### Bulk and scRNA-seq data acquisition and analysis

RNA sequencing was performed at the Columbia Sulzberger Genome Center. For bulk RNA-seq, total RNA from three independent biological replicates (naïve and ispinesib resistant cells) was isolated using the RNAqueous phenol-free total RNA isolation kit (Ambion, Life Technologies, Grand Island, NY) and DNA contamination in isolated RNA was removed by DNase treatment using TURBO DNA-free^™^ kit (Ambion, Life Technologies, CA). All samples had an RNA Integrity Number greater than 7.6, as assessed using Agilent Bioanalyzer. Libraries were prepared using the Illumina TruSeq RNA Library Prep Kit v2 and 20 million single-end, 100 base reads were acquired on an Illumina NovaSeq 6,000 sequencer. Reads were pseudoaligned to a kallisto mouse transcriptome index (GRCm38) using kallisto (0.44.0), and differential gene expression analysis was determined using DESeq2. Gene Set Enrichment Analysis (GSEA) was performed on the desktop version of GSEA (v4.1.0), using Hallmarks (Liberzon et al., 2015) and the Verhaak_Glioblastoma_mesenchymal gene sets from the Molecular Signatures Database (MSigDB).

Single cell RNA-seq (scRNA-seq) was performed using the 10x Genomics Chromium Single Cell 3’ Solution (v2 chemistry). Pooled 3’-end libraries were sequenced on an Illumina HiSeq 4000 sequencer. The resulting reads were aligned and demultiplexed using the 10x Genomics Cell Ranger pipeline. Marker selection, UMAP projections, clustering, and cluster-specific differential expression for each tumor was performed using the computational pipeline described in (Yuan et al., 2018)) and Mizrak et al. (2019) (https://github.com/simslab/cluster_diffex2018), using the raw transcripts count matrix. Data was then loaded into programming language R via the Seurat package 4.0.1 for downstream analysis and visualization.

Raw transcript counts were normalized in Seurat by with default parameters performing the transformation log_2_(counts per million + 1), and expression of select genes were projected on individual cells in the UMAP. Co-expression analysis was performed as follows: The transcript counts across the two conditions (naïve and resistant) were randomly subsampled to ensure that the coverage was the same across both conditions. Then, if a given cell expresses any number of transcripts of both Egfr and Src, was deemed double-positive (Egfr+/Src+), otherwise it was deemed negative. Then, a chi-squared test was performed between the fraction of double-positive cells between the resistant and naïve populations.

Differential gene expression analysis comparing ispinesib resistant and naïve cells was performed in Seurat using default parameters. Genes were ranked by log-2-fold change and Pre-ranked GSEA was performed comparing the two conditions for two MSigDB Cancer Hallmarks pathways (IL6_JAK_STAT3_SIGNALING and APOPTOSIS).

Cluster-specific differential expression analysis was then performed for each cluster to rank genes by log2-fold change and perform pre-ranked GSEA on the same two MSigDb Cancer Hallmarks pathways for each cluster to generate Normalized Enrichment Scores (NES) (Subramanian et al., 2005). NES were then projected on UMAP.

#### Pharmacokinetic studies of saracatinib

Equal numbers of male and female Wild-type and TKO FVB mice of age 8–14 weeks were used for all pharmacokinetic studies. Saracatinib dosing solution was formulated in pH 5.5 Sorensen’s phosphate buffer/25% (w/v) β-hydroxy cyclodextran to a final concentration of 1 mg/mL. A dose of 5 mg/kg saracatinib was administered via tail vein. Subsequently, mice were randomized into groups and n = 4 animals per time point were sacrificed at the following time points: 1, 2, 4, 8, 12, 16 and 24 h. At the time of sacrifice, mice were euthanized via CO_2_ and blood and brain were rapidly collected. Plasma was separated via centrifugation for 15 min at 7500 rpm, and tissues were frozen at −80°C until LC-MS/MS analysis. Prior to analysis, brain tissue was homogenized in 3X (w/v) 5% bovine serum albumin (BSA).

Saracatinib LC-MS/MS analysis was performed on a system consisting of a Waters Acuity HPLC connected to a Quattro Ultima LC-MS/MS in positive ion mode. Briefly, saracatinib was isolated from plasma and brain samples via liquid-liquid extraction using 5X volume of ethyl acetate and 2X volume pH 11 sodium hydroxide buffer. After vortexing and centrifugation, the supernatant was collected and dried completely under N_2._ Samples were reconstituted in mobile phase (MP), and 5 mL of each sample was injected onto a Phenomenex Synergi Polar-RP column (80Å, 75 × 2mm) for chromatographic separation at a flow rate of 0.5 mL/min. The LC method was isocratic (70:30, A:B) with a MP composition of A) H_2_O, 0.1% formic acid and B) acetonitrile, 0.1% formic acid and a total run time of 4 min. Dasatinib served as the internal standard, and the m/z transitions were as follows: saracatinib 542.33 → 127.13 and dasatinib 488.21 → 400.99. The standard curve was linear over the range of 1–1000ng/mL (weighted 1/Y^2^) with coefficients of variation less than 15%.

#### Phosphotyrosine sample processing and enrichment

Samples were lysed in 8M urea 1X HALT Protease/Phosphatase Inhibitor Cocktail (Thermo Scientific), reduced with 10mM dithiothreitol for 30 min at 56°C, alkylated with 55mM iodoacetamide for 45 min at room temperature (RT), and diluted 8-fold with 100mM ammonium acetate, pH 8.9. Proteins were subsequently digested with trypsin (Promega, sequencing-grade) at an enzyme to substrate ratio of 1:50 overnight at RT. Enzymatic activity was quenched with glacial acetic acid, and peptides were desalted and concentrated using C18 Sep-Pak cartidges (Waters). Peptides were eluted using 40% acetonitrile (MeCN) in 0.1% acetic acid, dried using vacuum centrifugation and lyophilized in 200 μg aliquots. Lyophilized peptides were labeled using tandem mas tag (TMT) 10-plex (Thermo Fisher Scientific) by resuspending lyophilized peptides in 70uL ethanol and 30uL of 0.5 triethylammoniumbicarbonate and incubated with TMT resuspended in 30uL anhydrous acetonitrile for 1 h at RT. Labeling reactions were quenched with a 15-min incubation with 0.3% of hydroxylamine. Samples were then pooled, dried by vacuum centrifugation, and stored at −80°C prior to analysis.

Phosphotyrosine enrichment of labeled samples was performed using a phospohotyrosine immunoprecipitation (IP) followed by immobilized metal affinity chromatography (IMAC). Briefly, labeled peptides were incubated with protein G agarose beads conjugated with 12 ug 4G10 (Millipore) and 9 ug PT-66 (Sigma) overnight at 4°C. Tyrosine phosphorylated peptides were eluted from beads for 30 min with 100 mM glycine, pH 2.5, and further enriched using IMAC with a column packed in house (200 uM ID X 10 cm packed with Poros 20 MC beads [Thermo]). Peptides were eluted using 250 mM NaH_2_PO_4,_ pH 8.0 onto a trapping column packed in house [100 μm ID × 10 cm packed with 10 μm C18 beads (YMC gel, ODS-A, 12 nm, S-10 μm, AA12S11)]. The column was then connected in series to an analytical capillary column prepared in house [50 μm ID X 10 cm packed with 5 μm C18 beads (YMC gel, ODS-AQ, 12 nm, S-5 μm, AQ12S05)] with an integrated electropspray tip.

Phosphopeptides were eluted using a 140 min gradient, 13–42% buffer B (70% Acetonitrile, 0.2 acetic acid) from 10–105 min and 42–60% buffer B from 105–115 min, 60–100% B from 115–122 min, and 100–0% B from 128–130 min at a flow rate of 0.2 mL/min on the HPLC with a flow split of approximately 10,000:1. Peptides were analyzed using a Thermo Fisher Q Exactive Plus Hybrid Quadrupole-Orbitrap mass spectrometer, and data was acquired using Thermo Fisher Scientific Xcalibur version 2.8.1.2806. Standard mass spectrometry parameters were as follows: spray voltage, 3.0 kV; no sheath or auxiliary gas flow; heated capillary temperature, 250°C.

The Q Exactive Plus was operated in data-dependent acquisition (DDA) mode with the following scan settings: Full-scan mass spectra (resolution 70k) were detected in the Orbitrap analyzer after accumulation of ions at 3 × 10^6^ target value with a maximum injection time (IT) of 350 ms. For every full scan, the top 15 most intense ions were isolated (isolation width of 0.4 m/z) and fragmented (collision energy (nCE): 33%) by higher energy collisional dissociation (HCD) with a maximum IT 350 ms, AGC target 1 × 10^5^, and 35k resolution. Unassigned and +1 charge states were excluded, and dynamic exclusion was set to 30 s. Crude peptide analysis was performed on a Q Exactive Plus mass spectrometer to correct for variation in peptide loadings across TMT channels.

Raw mass spectral data files were analyzed using Proteome Discoverer version 1.4 (Thermo Fisher Scientific) and searched against the human SwissProt database using Mascot version 2.4 (Matrix Science). Spectra were matched using an initial mass tolerance of 10 ppm on precursor masses and 15 mmu for fragment ions, with fixed modifications of cysteine carbamidomethylation, TMT-labeled lysine and TMT-labeled protein N-terminals. Variable modifications were oxidized methionine and serine, threonine, and tyrosine phosphorylation. Peptides were filtered using the following criteria: Ion score ≥20, ≥1 tyrosine phosphorylated residue, reporter ion quantification across all replicates. TMT reporter ions were added together across scans of the same pTyr peptide, and relative quantification was calculated as the log_2_ fold change for each sample over the average signal from the DMSO control. Hierarchical clustering of phosphosites was performed using Matlab R2019b.

#### Drug synergy

Synergy was calculated using the MuSyC algorithm as previously described (Wooten et al., 2021; Meyer et al., 2019). MuSyC quantifies two types of drug synergy, synergistic potency and synergistic efficacy, both relating to geometric transformations of the dose response surface, which are analogous to the transformations in the 1D Hill equation for potency (horizontal shift in the EC50) and efficacy (vertical shift in Emax). Synergy was calculated by fitting a dose-response surface relating the drug’s effect (rate of cell growth or static cell count at 96 h) to the concentrations of drug 1 and drug 2 (Figures 3D, 3E, and S3). To help the growth-rate-based fits converge, the bounds for E0 (effect with no drug) and E1, E2, E3 (maximal effects of drug 1, 2, and the combination) were set to [0.005, 0.002] and [0.01, −0.01], respectively. HSA synergy scores were calculated as described in Wooten et al. (2021).

### QUANTIFICATION AND STATISTICAL ANALYSIS

#### Statistical analysis

For *in vitro* studies, a two tailed t test or one-way ANOVA was used to calculate p values with statistical significance at p < 0.05. For survival studies, statistical significance was determined using a log rank test, and significance was set at p < 0.05.

## Supplementary Material

1

2

## Figures and Tables

**Table:** KEY RESOURCES TABLE

REAGENT or RESOURCE	SOURCE	IDENTIFIER

Antibodies		

Mouse monoclonal ant-Phospho-STAT3 (Tyr705) (M9C6)	Cell Signaling Technology	Cat# 4113 RRID: AB_2198588
Rabbit monoclonal ant-Phospho-STAT3 (Ser727)	Cell Signaling Technology	Cat# 9134 RRID: AB_331589
Mouse monoclonal anti-STAT3 (124H6)	Cell Signaling Technology	Cat# 9139 RRID: AB_331757
Rabbit polyclonal anti-AKT	Thermo Fisher Scientific	Cat# PA5-96351 RRID: AB_2808153
Rabbit polyclonal anti-Phospho-AKT (Ser 124)	Thermo Fisher Scientific	Cat# PA5-105161 RRID: AB_2816634
Rabbit polyclonal anti-Phospho-Src (Tyr418)	Millipore	Cat# 07-909; RRID: AB_568805
Rabbit polyclonal anti-Src	Cell Signaling Technology	Cat# 2108; RRID: AB_331137
Rabbit anti-Phospho-p70 S6 Kinase (Thr421/Ser424)	Cell Signaling Technology	Cat# 9204 RRID: AB_2265913
Rabbit anti-p70 S6 Kinase	Cell Signaling Technology	Cat# 9202 RRID: AB_331676
Rabbit monoclonal anti-Phospho-EGF Receptor (Tyr1068) (D7A5)	Cell Signaling Technology	Cat# 3777; RRID: AB_2096270
Rabbit polyclonal anti-EGF Receptor	Cell Signaling Technology	Cat# 2232; RRID: AB_331707
Mouse monoclonal anti-b-Actin (8H10D10)	Cell Signaling Technology	Cat# 3700; RRID: AB_2242334
Rabbit polyclonal anti-KIF15	Thermo Fisher Scientific	Cat# 55407-1-AP; RRID: AB_11182836
Rabbit monoclonal anti-α-Tubulin (11H10)	Cell Signaling Technology	Cat# 2125; RRID: AB_2619646
Rabbit monoclonal anti-COX IV (3E11)	Cell Signaling Technology	Cat# 4850; RRID: AB_2085424
Rabbit anti-Caspase-3	Cell Signaling Technology	Cat# 9662; RRID: AB_331439
Mouse monoclonal anti-DDDDK-Tag	MBL International	Cat#M185-3L; RRID: AB_11123930
Rabbit anti-Phospho-Histone H3 (Ser 10)	Cell Signaling Technology	Cat# 9701; RRID: AB_331535
Rabbit monoclonal anti-Histone H3 (D1H2)	Cell Signaling Technology	Cat#4499; RRID: AB_10544537

Bacterial and virus strains		

PDGF-IRES-Cre retrovirus	Lei et al. (2011); Kenchappa et al. (2020)	NA
pAdCMV/v5-DEST-S727D-STAT3-3xFlag plasmid	Addgene	Cat# 99263
pAdCMV/v5-DEST-S727A-STAT3-3xFlag plasmid	Addgene	Cat# 99262
STAT3-C Flag pRc/CMV	Addgene	Cat# 8722
pLenti Lifeact-EGFP BlastR plasmid	Addgene	Cat# 84383

Chemicals, peptides, and recombinant proteins		

PDGF-AA	Peprotech	Cat# 100-13A
hFGF (Human Fibroblast Growth factor )	R&D systems	Cat# 233-FB-025
hEGF (Human Epidermal Growth Factor)	Sigma-Aldrich	Cat# E9644
Fibronectin	Millipore-Sigma	Cat# FC010
Heparin	STEMCELL Technologies	Cat# 07980
N2 supplement	Gibco life technologies	Cat# 17502-048
NeuroPlex supplement	Gemini	Cat# 400-161
Ispinesib	Axon Medchem	Cat# 2446-25
SH5-07	Selleck Chemicals	Cat# S7923
Saracatinib	Selleck Chemicals	Cat# S1006
Dasatinib	Selleck Chemicals	Cat# S1021
Etoposide	Selleck Chemicals	Cat# S1225
Elacridar	Selleck Chemicals	Cat# S7772
Rhodamine Phalloidin	Cytoskeleton	Cat# PHDR1
VECTASHIELD with DAPI	Vector Laboratories	Cat# U-1500
Formaldehyde, 10% methanol free	CHEM (VWR)	Cat# 87001-890
SUPER signal West Pico PLUS Chemiluminescent substrate	Thermo Fisher Scientific	Cat# 34580
PBS	Thermo Fisher Scientific	Cat# 21-040
PVDF membrane	Biorad	Cat# 1620174
DMSO	Corning	Cat# 25-950-cqc
BSA	Thermo Fisher Scientific	Cat# 23209
Non-fat dry milk (Blotting grade blocker)	Biorad	Cat# 170-6404
Accutase	Sigma-Aldrich	Cat# A6964-500
Ethanol	Thermo Fisher Scientific	Cat# 61500-0020
Tween 20	Sigma-Aldrich	Cat# P1379
TBS	Thermo Fisher Scientific	Cat# 28358
Protease Inhibitor Cocktail, EDTA-free (100X)	Thermo Fisher Scientific	Cat# 87785
Pierce T-1step transfer buffer	Thermo Fisher Scientific	Cat# 84731
Western blot striping buffer	Thermo Fisher Scientific	Cat# 46430
10X Tris/Glycine/SDS buffer	Biorad	Cat# 1610772
Geltrex	Gibco	Cat# A14132-01
Laminin	Thermo Fisher Scientific	Cat# 3400-010-02
RIPA lysis buffer	Thermo Fisher Scientific	Cat# 89900
B27 supplement	Thermo Fisher Scientific	Cat# A3582801
Laemmli SDS Sample Buffer, reducing, 6X	Thermo Fisher Scientific	Cat# AAJ61337AC
Anti-Anti (100x)	Gibco	Cat# 15240-062
D-Luciferin	Perkin Elmer	Cat# 122799
Lipofectamine 3000 transfection agent	Life Technologies	Cat# 11668027
Doxorubicin	Selleck Chemicals	Cat# S1208
Blasticidine-S-Hydrochloride	Sigma-Aldrich	Cat# SBR00022
Kif15-IN-1	Medchem Express	Cat# HY-15948/CS-2697
Nocodazole	EMD Millipore	Cat# 487928

Critical commercial assays		

CellTiter-Glo 2.0 assay kit	Promega	Cat# G9242
Pierce^™^ BCA Protein Assay Kit	Thermo Fisher Scientific	Cat# 23225
RNAqueous^™^ Total RNA Isolation Kit	Thermo Fisher Scientific	Cat# AM1912
TURBO DNA-free^™^ Kit	Thermo Fisher Scientific	Cat# AM1907
Mitochondria Isolation Kit for cultured cells	Abcam	Cat# ab110170
Nuclear Extraction Kit	Abcam	Cat# ab113474

Experimental models: Cell lines		

Trp53^−/−^	This paper	NA
Trp53/Pten^−/−^	This paper	NA
GBM1A	(Galli et al., 2004)	NA
GBML1	(Deleyrolle et al., 2011)	NA
GBM612	Kenchappa et al. (2020)	NA
GBM108	Gift from Dr. Quiñones-Hinojosa	NA
GBM120	Kenchappa et al. (2020)	NA
MES1861	Reilly et al. (2000); Gürsel et al., 2011	NA
MES4622	Reilly et al. (2000); Gürsel et al., 2011	NA
PN20	Hambardzumyan et al. (2009)	NA
PN24	Hambardzumyan et al. (2009)	NA
PN62	Hambardzumyan et al. (2009)	NA

Experimental models: Organisms/strains		

Trp53^fl/fl^ mice	Jackson Laboratory	Stock# 008462
NOD-SCID mice	Jackson Laboratory	Stock# 005557
Wild type FVB mice	Taconic Biosciences Inc	NA
FVB mice	Taconic Biosciences Inc	NA

Software and algorithms		

Gene Set Enrichment Analysis (GSEA), hallmark analysis (version h.all.v7.0)	Broad Institute	http://software.broadinstitute.org/gsea/index.jsp

Deposited data		

Bulk and single cell RNA-seq data	Gene Expression Omnibus database	GSE193180 (all) GSE193178 (bulk RNA-seq) GSE193179 (scRNA-seq)
Mass spectrometry proteomics data	ProteomeXchange Consortium	PXZD030715https://doi.org/10.6019/PXD030715

## Highlights

- Resistance to mitotic kinesin inhibitors reduces their efficacy in treating cancer
- In GBM, resistance works through phosphorylation of STAT3 at Y705 and S727
- Resistance can be reversed by blocking phosphorylation with SRC and EGFR inhibitors
- Blocking resistance makes these drugs effective in pre-clinical GBM models
