# Supplementary material for: Activation of STAT3 through combined SRC and EGFR signaling drives resistance to a mitotic kinesin inhibitor in glioblastoma
Source: Cell Rep. Author manuscript; Available in PMC 2023 Mar 16. (PMC10018805; doi:10.1016/j.celrep.2022.110991)
Supplement: 1 [file NIHMS1818172-supplement-1.pdf]

Cell Reports, Volume *39*

## Supplemental information

# Activation of STAT3 through combined SRC and EGFR signaling drives resistance to a mitotic kinesin inhibitor in glioblastoma

**Rajappa S. Kenchappa, Athanassios Dovas, Michael G. Argenziano, Christian T. Meyer, Lauren E. Stopfer, Matei A. Banu, Brianna Pereira, Jessica Griffith, Afroz Mohammad, Surabhi Talele, Ashley Haddock, Natanael Zarco, William Elmquist, Forest White, Vito Quaranta, Peter Sims, Peter Canoll, and Steven S. Rosenfeld**

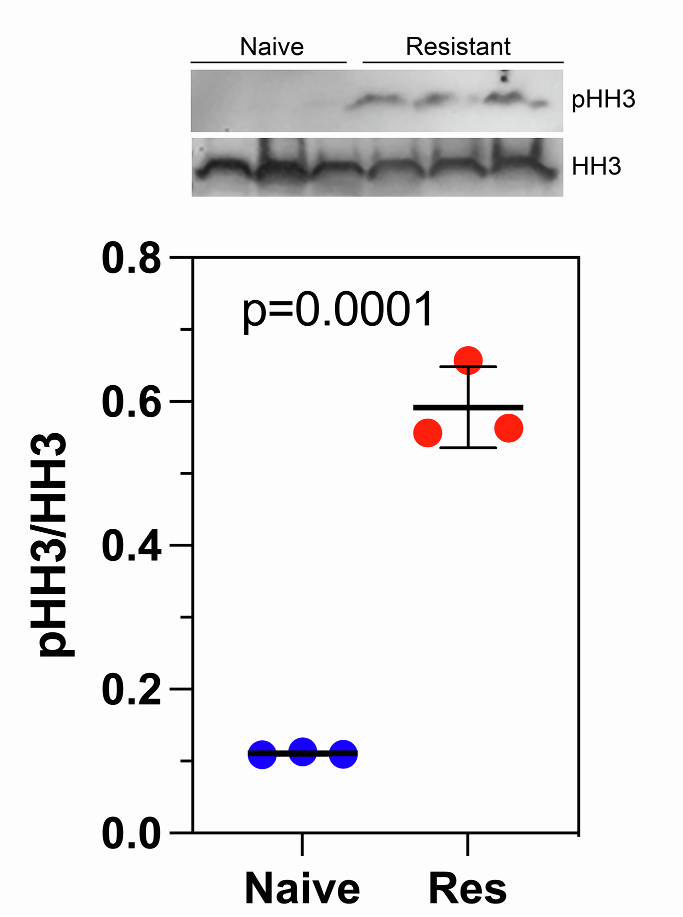


**Figure S1: Ispinesib resistant cells increase expression of phospho histone H3 (*pHH3*). Related to Fig. 1** Three biological replicates of ispinesib naïve (*Naïve*) and resistant (*Res*) cells, the latter maintained in the presence of 75 nM ispinesib, were probed on Western blot (*top*) for the expression of the mitotic marker pHH3. The ratio of pHH3 to total histone H3 (*HH3*, *bottom*) is significantly greater in ispinesib resistant cells (*p=0.0001, two tailed t test*), consistent with resistant cells experiencing a partial block in $G_2M$ with prolonged ispinesib treatment.

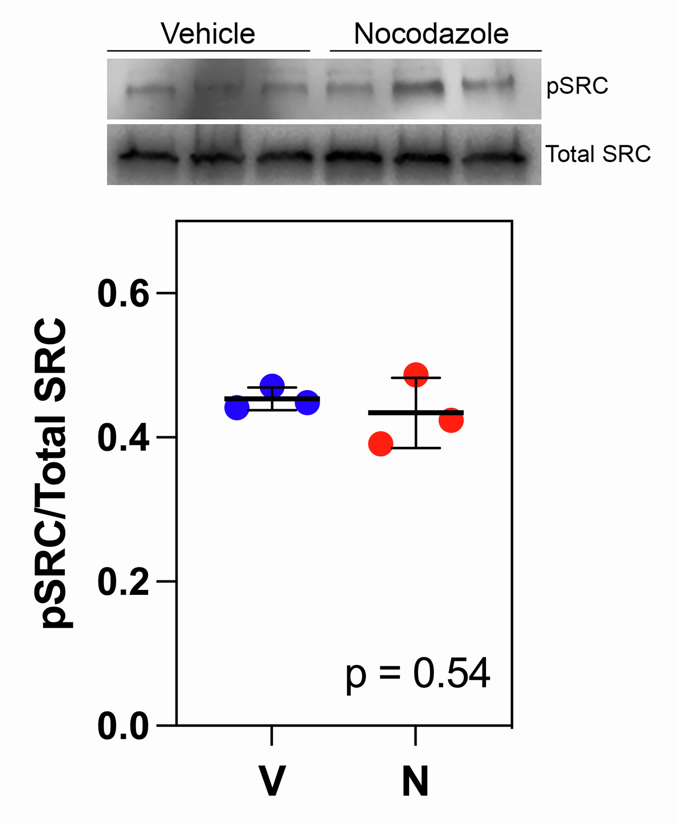


**Figure S2: Nocodazole does not increase SRC phosphorylation in *Trp53/Pten(-/-)* GBM cells. Related to Fig. 3.** Murine *Trp53/Pten(-/-)* GBM cells were treated with 100 nM nocodazole (*N*) or vehicle (*DMSO*, *V*) for 24 hours, and cell lysates were probed for total and pY418 SRC. The content of pY418 SRC, normalized to total SRC, is unchanged by treatment with nocodazole (*p=0.54, two tailed t test*).

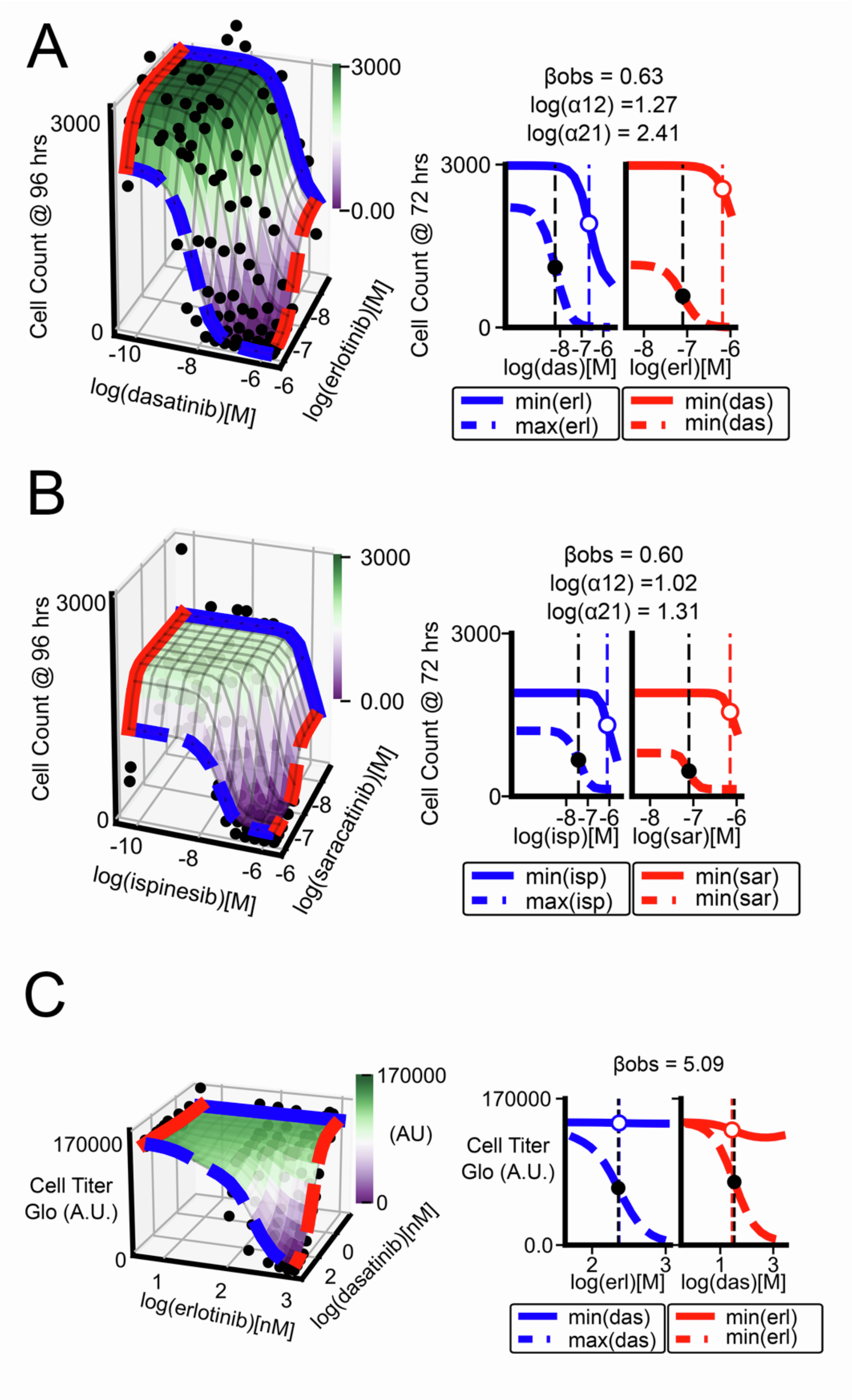


**Figure S3: MuSyc analysis of synergy using static cell counts. Related to Fig. 3D, E and Fig. 5I, J. (A)**. (*Left*) Dose-response surface for the combination of dasatinib and erlotinib for ispinesib-resistant *Trp53/Pten(-/-)* cells cultured in the presence of 75 nM ispinesib, using manual cell count after 96 hours of exposure to drug. This surface, based on cell number, is analogous to the left panel in **Fig. 3D**, which is based on cell doubling time. (*Right*) Projected edges of the surface in **Fig. S1A** for the maximum and minimum tested in the combination, analogous to the right panel in **Fig. 3D**. **(B).** (*Left*) Dose-response surface for the combination of ispinesib and saracatinib ispinesib-resistant

*Trp53/Pten(-/-)* cells, using manual cell count after 96 hours of exposure to drug. This surface, based on cell number, is analogous to the left panel in **Fig. 3E**, which is based on cell doubling time. (*Right*) Projected edges of the surface in **Fig. S1B** for the maximum and minimum tested in the combination, analogous to the right panel in **Fig. 3E**. **(C).** Corresponding dose response surface for the combination of dasatinib and erlotinib for an ispinesib resistant human GBM cell line (*L1*). Consistent with **Fig. 5I, J**, neither dasatinib nor erlotinib have any appreciable activity as single agents against ispinesib-resistant L1 cells, making calculations of $EC_{50}$ highly uncertain. However, the dose response surface shows a marked degree of synergy of efficacy ($\beta_{obs} = 5.09$).

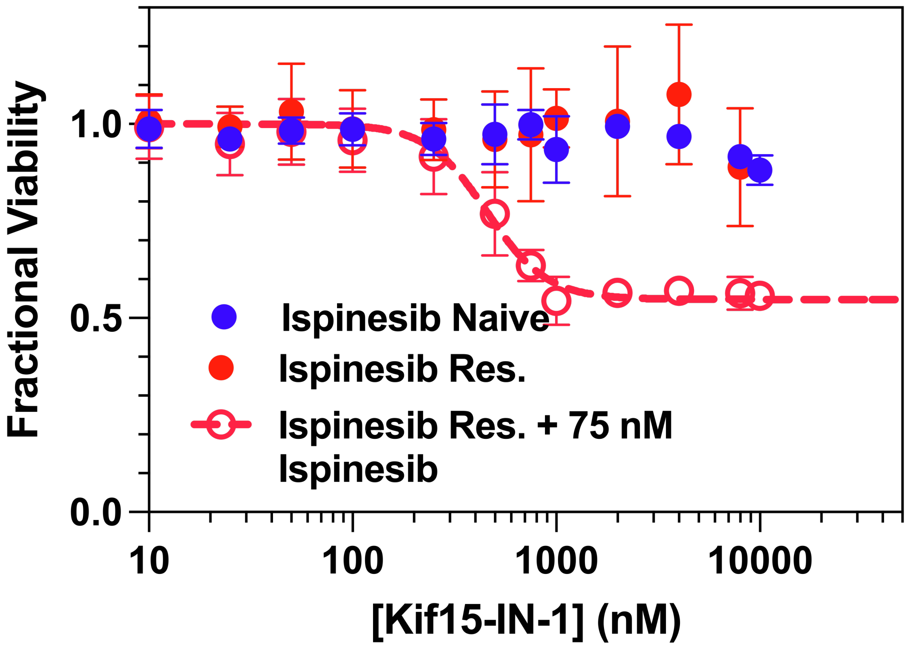


**Figure S4: Kif15-IN-1 reduces viability of ispinesib resistant cells in the presence of ispinesib. Related to Fig. 4.** Murine *Trp53/Pten*(-/-) cells that are ispinesib naïve or resistant (*Res.*) in the absence of ispinesib were treated in vitro with a range of doses of the Kif15 inhibitor Kif15-IN-1 and cell viability was measured after 3 days of exposure. Neither cell type shows any appreciable sensitivity to Kif15-IN-1. However, in the presence of 75 nM ispinesib, Kif15-IN-1 reduces cell count by 45% with an $EC_{50}$ of 456 ± 52 nM (**Table S1**)

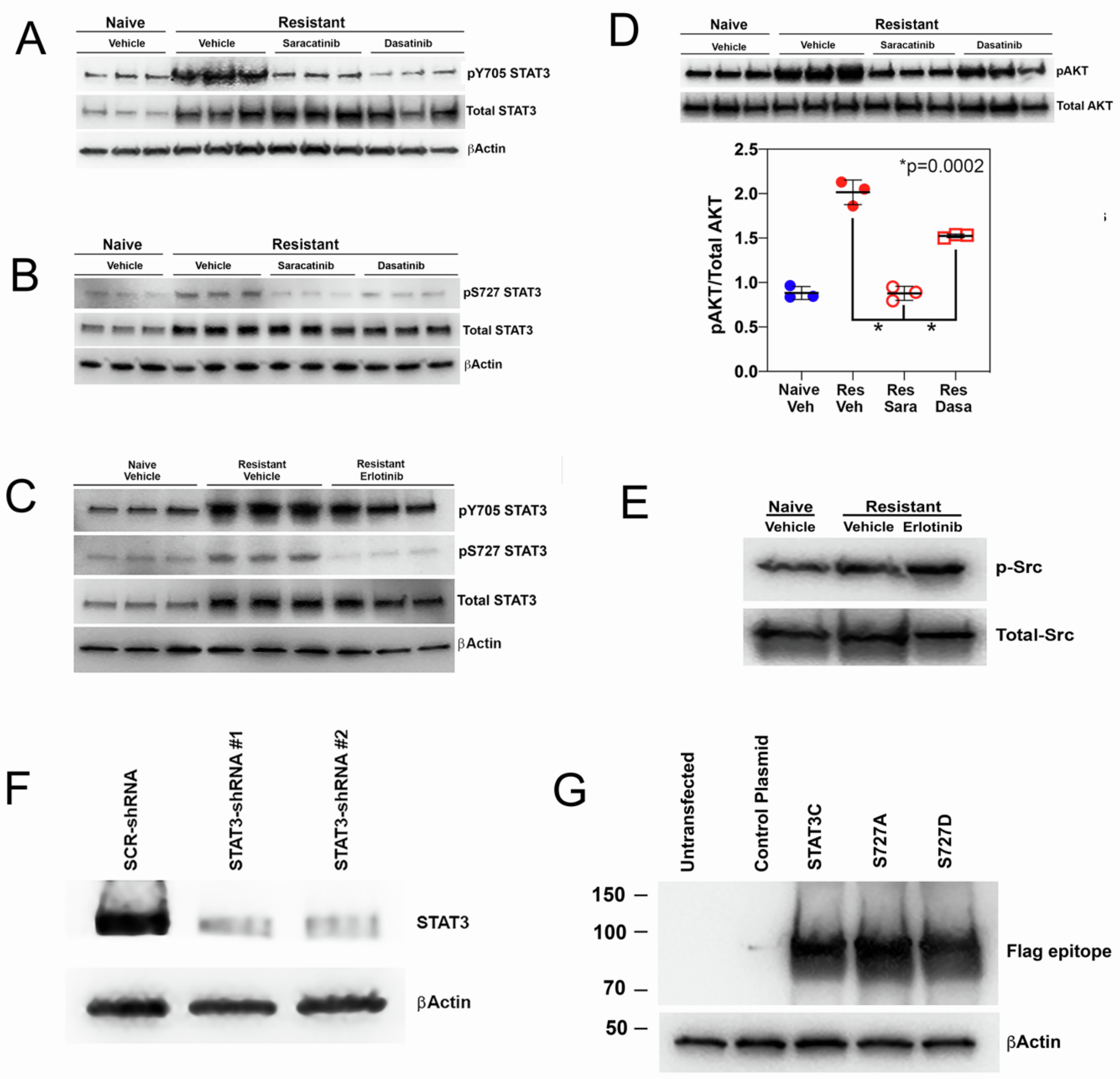


**Figure S5: Phosphorylation of STAT3 at Y705 and S727 contribute to ispinesib resistance in murine *Trp53/Pten(-/-)* cells. Related to Fig. 4. (A).** Western blots of lysates from ispinesib naïve and resistant cells, probed for total and pY705 STAT3. Ispinesib resistance is associated with increased expression of both total STAT3 and pY705 STAT3. Treatment of resistant cells with either saracatinib or dasatinib reduces pY705 STAT3 levels to those of naïve cells. Please refer to **Fig. 4E** & **G** for quantitation. **(B).** Corresponding Western blots probing for pS727 STAT3. Please refer to **Fig. 4F** for quantitation. **(C).** Western blots of lysates from ispinesib naïve and resistant cells, probed for total and pY705 and pS727 STAT3. Treatment of resistant cells with erlotinib markedly reduces levels of pS727 STAT3 but has relatively little effect on levels of pY705 STAT3. Please refer to **Fig. 4H-J** for quantitation. **(D).** Western blots of lysates from ispinesib naïve and resistant cells, probed for total and phosphorylated AKT (*Top*). Treatment of ispinesib resistant cells with saracatinib reduces pAKT levels to those of naïve cells treated with vehicle, while treatment with dasatinib does not (*Bottom*). **(E).** Treatment of ispinesib resistant cells with erlotinib does not appreciably alter levels of SRC phosphorylation. **(F).** Western blot demonstrating >95% suppression of STAT3 in ispinesib resistant cells with shRNA. Related to **Fig. 4L** & **M**. **(G).** Ispinesib naïve cells were transfected with vectors encoding STAT3-C, S727A STAT3 or S727D STAT3 constructs, each fused to a Flag

epitope. Probing of lysates from transfected cells demonstrates staining of the Flag epitope at the expected $M_r$ for the STAT3-Flag fusion (*89.1 kDa*).

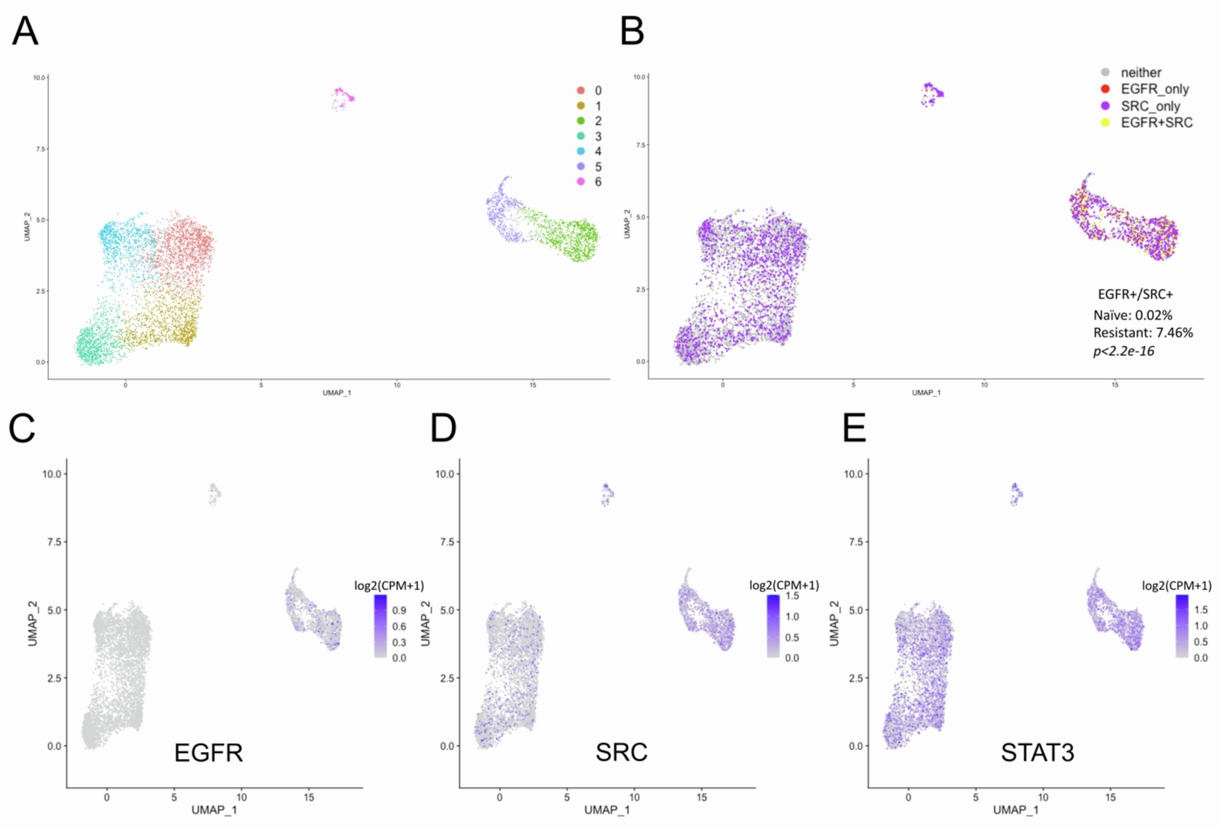


**Figure S6: Single cell RNA-seq studies of ispinesib naïve and resistant *Trp53/Pten(-/-)* cells. Related to Fig. 6. (A).** UMAP projection annotating cells by cluster designation as described in the methods. **(B).** Co-expression analysis of Egfr and Src transcripts on randomly subsampled dataset to equalize transcript coverage across conditions. Cells were deemed Egfr or Src positive based on whether they express >0 transcripts of the respective gene. (**C-E).** Projecting normalized gene expression (*log2(CPM+1)*) of *Egfr* (**C**), *Src* (**D**), and *Stat3* (**E**) onto each cell on the UMAP.

## Table S1: Dose Response Parameters Related to Figures 1-5 and S3[a]

| Cell Line | Drug | $EC_{50}$ (nM) | n | c | Figure |
|---|---|---|---|---|---|
| *Trp53/Pten(-/-)* Naive | Ispinesib | 3.9 ± 0.3 | 0.4 | 1 | 1A |
| *Trp53(-/-)* Naive | Ispinesib | 28.1 ± 0.5 | 0.5 | 1 | 1A |
| *Trp53(-/-)* Resistant | Ispinesib | 2895 ± 953 | 1.0 | 1 | 1A |
| | | | | | |
| 120 Naïve | Ispinesib | 10.3 ± 1.6 | 0.3 | 1 | Q |
| 612 Naïve | Ispinesib | 5.9 ± 1.2 | 0.4 | 1 | 1B |
| L1 Naïve | Ispinesib | 11.5 ± 1.9 | 0.5 | 1 | 1B |
| | | | | | |
| *Trp53/Pten(-/-)* Resistant | Elacridar | 2977 ± 312 | 0.9 | 1 | 2E |
| *Trp53/Pten(-/-)* Resistant +Ispinesib | Elacridar | 1503 ± 178 | 1.0 | 1 | 2E |
| | | | | | |
| *Trp53/Pten(-/-)* Resistant + 75 nM Ispinesib | SH5-07 | 99.8 ± 14.7 | 0.7 | 1 | 4K |
| *Trp53/Pten(-/-)* Resistant | SH5-07 | 1634 ± 116 | 1.3 | 1 | 4K |
| *Trp53/Pten(-/-)* Naive | SH5-07 | 3435 ± 210 | 1.9 | 1 | 4K |
| | | | | | |
| *Trp53/Pten(-/-)* Naïve S727A transfected | Ispinesib | 17.2 ± 1.8 | 0.5 | 1 | 4Q |
| *Trp53/Pten(-/-)* Naïve S727D transfected | Ispinesib | 318.7 ± 23.2 | 0.8 | 1 | 4Q |
| *Trp53/Pten(-/-)* Naïve STAT3-C transfected | Ispinesib | 397 ± 30.1 | 1.1 | 1 | 4Q |
| *Trp53/Pten(-/-)* Naïve STAT3-C + S727D co-transfected | Ispinesib | 3174 ± 951 | 0.9 | 1 | 4Q |
| | | | | | |
| L1 Resistant + Ispinesib | Saracatinib | 73.7 ± 7.6 | 0.9 | 1 | 5K |
| 120 Resistant + Ispinesib | Saracatinib | 82.1 ± 6.6 | 1.2 | 1 | 5K |
| | | | | | |
| L1 Resistant | SH5-07 | 821 ± 53 | 1.8 | 1 | 5L |
| 120 Resistant | SH5-07 | 654 ± 33 | 1.6 | 1 | 5L |
| | | | | | |
| *Trp53/Pten(-/-)* Resistant + 75 nM Ispinesib | Kif15-IN-1 | 456 ± 52 | 2.9 | 0.45 | S4 |

[a]Dose response data were fit to a modified Hill equation:

$$f = 1 - \left( \frac{c}{1 + \left[ \frac{EC_{50}}{d} \right]^n} \right)$$

where f is the fraction of cells viable at drug concentration d, n is the Hill coefficient, and 1-c is the extrapolated fraction of cells remaining at infinite drug concentration.

**Table S3: Genes Contributing to the Core Enrichment of the Hallmark IL6_JAK_STAT3 Signaling and Apoptosis Gene Sets. Related to Figure 2.**

| HALLMARK_IL6_JAK_STAT3_SIGNALING_signal | |
|---|---|
| Description | Rank metric score |
| CSF1 | 3.127344131 |
| MAP3K8 | 2.989305019 |
| CXCL1 | 2.985844612 |
| PIM1 | 2.876420736 |
| CD14 | 2.684521437 |
| STAT2 | 2.472597837 |
| ACVR1B | 2.427387714 |
| CXCL10 | 2.319077969 |
| TLR2 | 2.197650909 |
| TNFRSF1B | 2.188010693 |
| IRF1 | 2.058646917 |
| OSMR | 2.02555275 |
| IL13RA1 | 1.996367693 |
| IL15RA | 1.889028072 |
| IFNGR2 | 1.88274014 |
| CCL7 | 1.850557685 |
| TYK2 | 1.831244469 |
| HMOX1 | 1.763247371 |
| SOCS3 | 1.72180295 |
| MYD88 | 1.545367479 |
| IRF9 | 1.529366493 |
| FAS | 1.476338387 |
| CXCL11 | 1.441642642 |
| IL1R1 | 1.438171387 |
| HAX1 | 1.386450648 |
| IL10RB | 1.168225765 |
| IL6 | 1.156022787 |
| PIK3R5 | 1.139425397 |
| PF4 | 1.098421574 |
| TGFB1 | 1.068340659 |
| PDGFC | 1.054469585 |
| IFNGR1 | 1.010493636 |
| CNTFR | 0.990882218 |
| IL1R2 | 0.983135819 |
| CXCL13 | 0.95810616 |
| CSF2 | 0.939159274 |
| STAM2 | 0.890168846 |
| IFNAR1 | 0.857622743 |
| CXCL3 | 0.838460088 |

| HALLMARK_APOPTOSIS_signal | |
|---|---|
| Description | Rank metric score |
| GADD45A | 3.051753759 |
| PMAIP1 | 2.99844861 |
| GCH1 | 2.748230696 |
| CD14 | 2.684521437 |
| BID | 2.377319813 |
| PDGFRB | 2.262815952 |
| ENO2 | 2.24559164 |
| BCL2L11 | 2.229264975 |
| BMF | 2.187267065 |
| BIRC3 | 2.071017981 |
| HGF | 2.069895744 |
| IRF1 | 2.058646917 |
| BTG3 | 2.015978575 |
| ISG20 | 2.012355566 |
| ATF3 | 2.004239559 |
| SMAD7 | 1.851454735 |
| IGF2R | 1.844976425 |
| HSPB1 | 1.791835904 |
| HMOX1 | 1.763247371 |
| IFITM3 | 1.758444548 |
| PLCB2 | 1.709822178 |
| DDIT3 | 1.604860544 |
| PDCD4 | 1.563193083 |
| GSTM1 | 1.562746763 |
| BTG2 | 1.508926868 |
| MGMT | 1.482244611 |
| FAS | 1.476338387 |
| GADD45B | 1.467140794 |
| BNIP3L | 1.461665988 |
| TAP1 | 1.447855949 |
| DPYD | 1.430681109 |
| SATB1 | 1.376746655 |
| SPTAN1 | 1.285884976 |
| IL1A | 1.263474345 |
| F2R | 1.249000192 |
| TIMP2 | 1.187370062 |
| IL6 | 1.156022787 |
| CLU | 1.146504879 |
| DCN | 1.100628495 |
| SOD2 | 1.099037886 |
| IFNB1 | 1.037020564 |
| TXNIP | 1.010776758 |
| IFNGR1 | 1.010493636 |
| TGFBR3 | 1.005265236 |
| CDKN1B | 0.989551544 |
| GNA15 | 0.968944073 |
| GPX4 | 0.915690064 |
| PPT1 | 0.899199188 |
| AIFM3 | 0.897068858 |
| IL18 | 0.85111165 |
| CCND2 | 0.829757333 |
| CASP6 | 0.794056118 |
| RELA | 0.76679492 |
| PAK1 | 0.742235661 |
| IER3 | 0.711185098 |
| IGFBP6 | 0.70779264 |
| PPP2R5B | 0.70758605 |
| TNFSF10 | 0.701559782 |
| BCL10 | 0.696632087 |
| MMP2 | 0.681311667 |
| SC5D | 0.671013474 |

**Table S4: Receptor Tyrosine Kinases Upregulated in Ispinesib Resistant Murine *Trp53/Pten(-/-)* GBM Cells. Related to Figure 2.**

| | baseMean | log2FoldChange | lfcSE | stat | pvalue | padj |
|---|---|---|---|---|---|---|
| **Ddr2** | 9392.264714 | 0.390455228 | 0.146879204 | 2.658342483 | 0.007852605 | 0.02413204 |
| **Fgfr1** | 5302.72259 | 0.351221519 | 0.147232045 | 2.385496439 | 0.017056087 | 0.046289903 |
| **Flt3** | 8.713994578 | 3.113619813 | 1.144913642 | 2.719523724 | 0.006537601 | 0.020681487 |
| **Flt4** | 28.70890712 | 1.698501414 | 0.63262833 | 2.684832994 | 0.007256606 | 0.022587646 |
| **Insr** | 1423.927513 | 0.589879312 | 0.22157298 | 2.662234864 | 0.00776237 | 0.023907958 |
| **Mertk** | 448.3537726 | 1.033627033 | 0.224681657 | 4.600406844 | 4.22E-06 | 3.33E-05 |
| **Pdgfrb** | 3367.937553 | 1.407861522 | 0.14692621 | 9.58209924 | 9.51E-22 | 5.58E-20 |
| **Ret** | 18.44404784 | 3.355623884 | 0.692048633 | 4.84882669 | 1.24E-06 | 1.10E-05 |
| **Ryk** | 4830.402374 | 0.488899581 | 0.122874072 | 3.978866908 | 6.92E-05 | 0.000401032 |
| **Tyro3** | 984.72402 | 0.753419505 | 0.184421462 | 4.085313594 | 4.40E-05 | 0.000269336 |
| **Epha1** | 66.9367502 | 4.455549255 | 0.652102274 | 6.83259273 | 8.34E-12 | 1.78E-10 |
| **Epha4** | 124.1048089 | 1.928074552 | 0.707799258 | 2.724041501 | 0.00644884 | 0.020475532 |
| **Epha3** | 28.69936978 | 1.487608868 | 0.471159533 | 3.157335812 | 0.001592179 | 0.006225962 |
| **Ephb4** | 1670.684477 | 0.526295371 | 0.140959614 | 3.733660698 | 0.000188717 | 0.000972843 |
| **Ephb6** | 382.9903593 | 1.155855686 | 0.463638309 | 2.493011605 | 0.012666471 | 0.036098351 |
| **Egfr** | 341.7866384 | 1.593361822 | 0.43508276 | 3.662203991 | 0.000250055 | 0.001244486 |

**Table S5: Pharmacokinetic Parameters for Saracatinib in Wild Type and TKO Mice. Related to Figure 7.**

| Parameter | Wild Type | TKO |
|---|---|---|
| Plasma $AUC_{0\rightarrow\infty}$ (hr·ng/ml) | 5111 | 4770 |
| Brain $AUC_{0\rightarrow\infty}$ (hr·ng/ml) | 1207 | 36185 |
| Brain $t_{1/2}$ (hr) | 3.8 | 6.8 |
| Cl (l/hr/kg) | 0.98 | 1.0 |
| $K_p$ ($AUC_{Brain}/AUC_{Plasma}$) | 0.24 | 7.72 |
